# Perceptual distortions in PredNet and quantification of top-down/bottom-up flow

**DOI:** 10.3389/fncom.2026.1869519

**Published:** 2026-07-07

**Authors:** Hiroki Kojima, Keisuke Suzuki, Yuichi Yamashita

**Affiliations:** 1 Department of Information Medicine, National Institute of Neuroscience, National Center of Neurology and Psychiatry, Tokyo, Japan; 2 Center for Human Nature, Artificial Intelligence, and Neuroscience (CHAIN), Hokkaido University, Sapporo, Hokkaido, Japan

**Keywords:** aberrant precision, HSIC, perceptual distortion, predictive coding, PredNet

## Abstract

Perceptual distortions are widely observed in various psychiatric diseases, including Autism Spectrum Disorder (ASD). Recent Bayesian models of psychiatric disorders and learning disabilities propose a general theory grounded on the concept of “aberrant precision.” However, these models have yet to be used as phenomenological models for visual distortions because previous models usually only deal with a low-dimensional input with Laplace approximation. Such assumptions are necessary for precision to be well-defined; otherwise, the explanation based on aberrant precision was hardly applicable. This study addresses these limitations using the predictive coding-based deep neural network PredNet and a new analysis method inspired by the precision account using the Hilbert-Schmidt Independence Criterion (HSIC). We found that the visual distortion happened when trained with more extended temporal contexts, which was mitigated when we increased the weight of the prediction error of the top layer. From HSIC analysis, we showed that this weight increase enhanced the top-down information flow in prediction, which led to an enhanced ability to capture global features of the visual input, such as rotation and average brightness and hue.

## 1 Introduction

Perception can be distorted even when sensory organs function normally. For example, photophobia–which increases sensitivity to light and induces avoidance behaviors–can develop due to impairments in the central nervous system (Katz and Digre, 2016; Noseda et al., 2019). Additionally, mental disorders can also lead to perceptual abnormalities. In schizophrenia, low contrast sensitivities and color vision impairments have been observed (Shoshina and Shelepin, 2015; Butler et al., 2008; Fernandes et al., 2019). Similarly, autism spectrum disorder (ASD) exhibits not only social symptoms but also visual symptoms, such as visual hypersensitivities (Parmar et al., 2021; Nagai et al., 2015) and difficulty in capturing global motions (Simmons et al., 2009; Robertson and Baron-Cohen, 2017).

Elucidating the underlying mechanisms of visual distortions through biological research alone is challenging. Therefore, computational accounts have been pursued to provide a macroscopic description of the phenomena. One of the main approaches in this direction is the theories of predictive processing (Rao and Ballard, 1999; Friston, 2005; Clark, 2013). Predictive processing is a general brain theory that explains that the brain works to minimize prediction errors, claiming that every brain function can be framed as an inference process. The inference process is usually formulated as Bayesian inference, where predictions are produced from the combination of prior belief (top-down information) and sensory inputs (bottom-up information). The relative importance of each information source in making predictions is called “precision” (Friston, 2005).

Precision has been assumed to be important in explaining psychiatric disorders (Adams et al., 2013; Lawson et al., 2014; Palmer et al., 2017; Chrysaitis and Seriès, 2023). They claimed that psychiatric conditions can arise from an imbalance in the integration of top-down and bottom-up information to produce predictions. This explanation, based on the misallocation of relative importance (precision) of these information sources, leads to what is known as the “aberrant precision” hypothesis. Based on this idea, some computational models have been proposed (Adams et al., 2013), and the extension of this type of model by adding a hierarchical structure or allowing precision adaptive has been attempted (Mathys et al., 2011, 2014). However, these models are limited because they only deal with low-dimensional inputs, whereas actual visual input is inherently high-dimensional data.

This study investigated whether we can model visual distortions using this predictive processing-based approach. Primarily, we aimed to model the phenomenological feature of visual distortions, following a series of studies under “computational phenomenology.” Computational phenomenology seeks to expand computational models to encompass lived experience in the philosophical tradition of phenomenology (Ramstead et al., 2022). Building on this philosophical idea, researchers have developed computational models to simulate perceptual characteristics–such as those observed in visual hallucinations–rather than focusing solely on objective cognitive and perceptual abilities (Suzuki et al., 2017; Schartner and Timmermann, 2020; Suzuki et al., 2024). In the present work, we aimed to apply this idea to visual distortions often observed in those mental or developmental disorders. This requires a computational model capable of reproducing features of subjective visual experience. Since the models widely used in the field of predictive processing only deal with low-dimensional input, they are insufficient for modeling the high-dimensional visual data. To overcome this limitation, we turned to a deep neural network model called “PredNet” (Lotter et al., 2016).

PredNet is a video prediction system that predicts the next frame of the video based on the past frames, and the architecture was inspired by the idea of predictive processing (Keller and Mrsic-Flogel, 2018; Millidge et al., 2021). The prediction process is driven only by prediction errors and has a hierarchical structure, so in this sense, PredNet is a hierarchical predictive processing system. Because of this similarity, PredNet has been used as a perception model in several studies. For example, Lotter et al. (2020) reported that its dynamics exhibit similarities to biological neurons, and Watanabe et al. (2018); Kobayashi et al. (2022) claimed that PredNet can even replicate visual motion illusions.

Based on these results, we decided to explore the possibility of PredNet to model visual distortions. Considering that visual distortions may arise from altered development of the predictive processing system, we prepared two developmental conditions that might lead to visual distortions: higher layer loss and state resetting, corresponding to the change in spatial context and temporal context, respectively. This state resetting condition refers to the duration of temporal dependencies in the predictive process. In hierarchical predictive systems like PredNet, predictions are influenced not only by immediate inputs but also by temporal memory stored within the network's architecture. By manipulating the availability of this temporal context, we aimed to understand how different lengths of temporal dependencies impact the network's performance. While longer temporal contexts can improve predictive accuracy by incorporating extended information, they are also known to introduce instability in the system (Parisi et al., 2019). Notably, the length and updating speed of temporal contexts have been implicated in the atypical processing observed in Autism Spectrum Disorder (ASD) (Lieder et al., 2019), making this an essential factor to explore in computational models of perception. By training the network under these different developmental conditions, we aimed to determine which manipulations could reproduce phenomenological features of visual distortions.

We analyzed the underlying mechanism that led to these outputs based on the predictive processing perspective, especially considering the concept of “aberrant precision” (Adams et al., 2013; Lawson et al., 2014; Palmer et al., 2017). However, applying this idea to the analysis of the PredNet model is not straightforward, mainly because there is no established method to quantify the ratio of top-down/bottom-up information used for prediction in PredNet. This issue did not exist in previous studies because the models explicitly included a “precision” parameter representing the balance between top-down and bottom-up information. In contrast, PredNet lacks such a parameter, so we must estimate this balance indirectly from the model's outputs. For this purpose, we introduced a new way to measure the relative importance of top-down/bottom-up information in making predictions. This method is based on the measure called the Hilbert-Schmidt Independence Criterion (HSIC) (Sugiyama and Yamada, 2012; Wu et al., 2018). HSIC measures the non-linear correlation between two random variables, making it suitable for high-dimensional data without resorting to Laplace approximations. Using this measure, we quantified the relative importance of top-down/bottom-up information to predictions.

Here is an overview of our paper: We trained PredNet under different developmental conditions by varying the weight of the highest layer loss λ and state resetting, simulating altered development of the predictive processing system. We then analyzed the predicted images by phenomenologically evaluating them to assess how well the model reproduces visual distortions. Subsequently, we utilized HSIC to gauge the contribution of top-down/bottom-up information to predictions. Lastly, we focused on a network trained with the resetting condition, exploring how the weighting of the top layer influenced output images and responses to perturbations.

## 2 Materials and methods

### 2.1 PredNet

As a model of a hierarchical deep predictive neural network, we used PredNet, proposed by Lotter et al. (2016).

This network as a whole learns to predict the next frame image of a video based on the preceding frames. Every time step *t*, the network first calculates the error *E*_0_ between the prediction $\hat{A_{0}}\left(t-1\right)$ and actual video frame *A*_0_(*t*), and the following prediction process, only this error is utilized, based on the idea from predictive coding.

**Figure 1:**
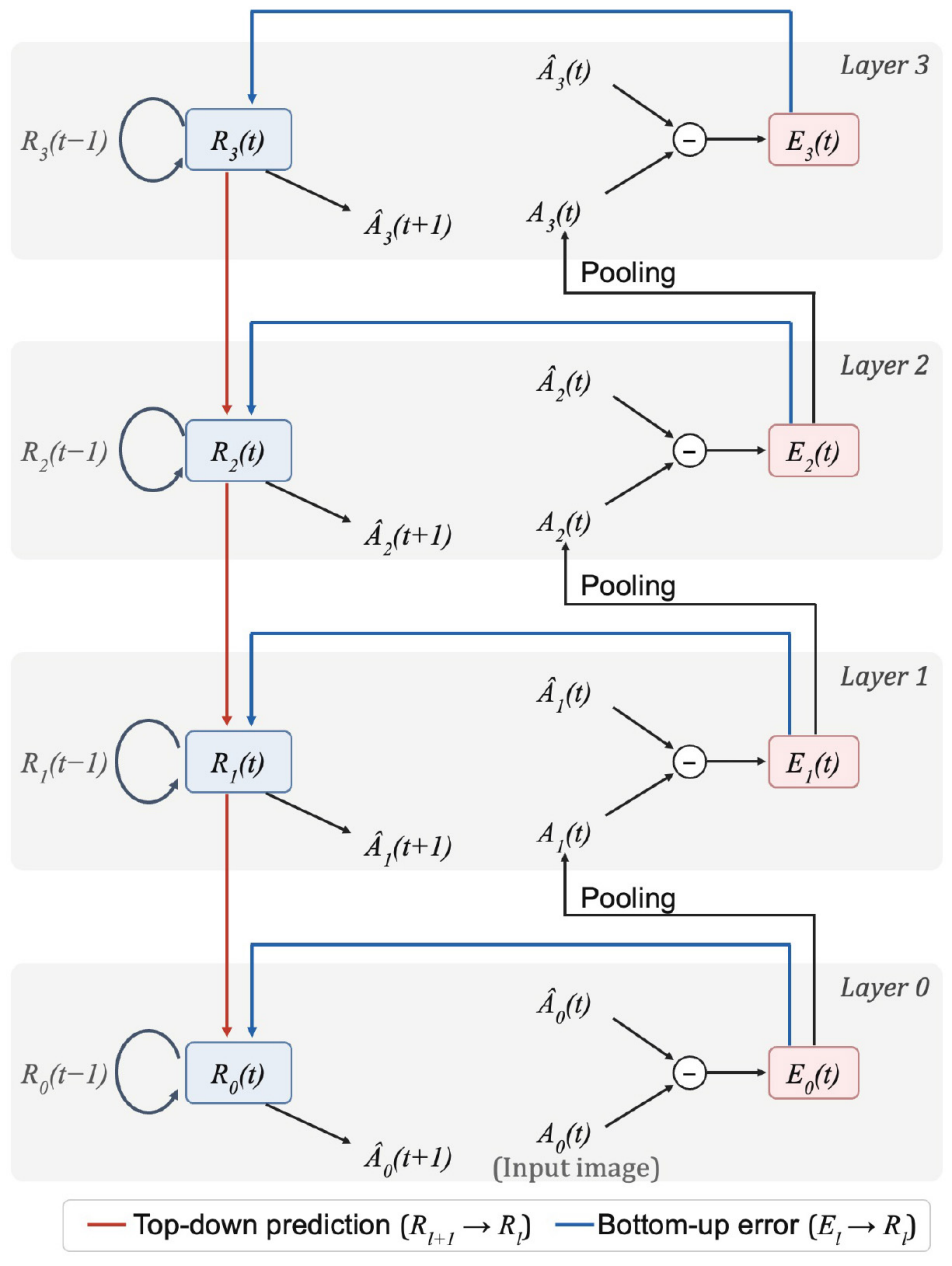
Four-layer architecture of PredNet used in the present study. Blue arrows indicate bottom-up information flow through error units, and red arrows indicate top-down information flow from higher-layer representations. Each layer contains convolutional and ConvLSTM units. Bottom-up transfer involves pooling.

Internally, this network is constructed hierarchically and consists of multiple layers (Figure 1). Here, we used the network with four layers (*l* = 0, 1, 2, 3). Each layer *l* includes a convolutional network and convolutional LSTM (ConvLSTM) and receives input both from the lower layer (*l*−1) and the higher layer (*l*+1). The lowest layer receives the error between the video frame and the prediction instead of the lower layer input, and the topmost layer does not receive the information from the higher layer. When the information is transferred from the lower layer to the upper layer, a 2 × 2 pooling operation is applied, which means that the higher layer deals with more spatially coarse-grained information.

The training loss function is defined as:


$$\begin{array}{l} L_{\text{train}}=\underset{t}{\sum }\underset{l}{\sum }{\text{λ}}_{l}E_{l}^{t}, \end{array}$$

where *l* is the index of each layer (*l* = 0, 1, 2, 3), and $E_{l}^{t}$ is the mean squared error at layer *l* at time *t*. λ_*l*_ denotes the loss weight for the prediction error at layer *l*. In all experiments, we fixed λ_0_ = 1 and λ_1_ = λ_2_ = 0, and manipulated only the highest-layer loss weight λ_3_. Specifically, we compared three values, λ_3_ ∈ {0, 1, 100}. Thus, the training loss used in the present experiments can be written as


$$L_{\text{train}}=\underset{t}{\sum }\left(E_{0}^{t}+{\text{λ}}_{3}E_{3}^{t}\right).$$

In this study, we adopted the implementation of PredNet by Watanabe et al. (2018) using Chainer (Tokui et al., 2015). The significant difference between this version and the original PredNet is the number of batches and the length of each input. In the original PredNet, each input video consisted of 10 frames and was trained with four batches. On the other hand, the implementation by Watanabe et al. (2018) used no batch training and kept inputting the same long video, while the weight update by backpropagation through time (BPTT) happened every 20 time steps.

This difference in the length of the input images also accompanied the difference in the resetting interval of the system's internal state, which is encoded in the ConvLSTM. When we start to feed the sequence of images, the internal state is reset. Therefore, in the original PredNet, the internal state resetting happens every 10 frames. In contrast, in the Watanabe et al. (2018) system, the resetting of the internal state does not happen during the training because the input consists of a single long video.

We treated this resetting interval as an operational manipulation of state-mediated temporal context, because resetting or carrying over ConvLSTM internal states changes how temporal information is retained during training. We prepared two conditions: a “no-reset condition,” in which the network was trained without periodic state resetting, and a “reset condition,” in which the internal state was reset at the time of weight updating.

### 2.2 Dataset

As a training dataset, we used the First-Person Social Interactions Dataset (Fathi et al., 2012) modified by Watanabe et al. (2018). We further applied two modifications to the dataset, which are downsampling to 15 frames per second (fps) from 30fps and cropping from (160, 120) to (160, 80). Our dataset consists of 200,000 images, and we trained the network up to 200,000 iterations.

### 2.3 Experimental conditions

We compared the PredNet by changing the two developmental conditions described below. The first training parameter is the weighting of higher layer error in the loss function. In PredNet, the higher layer deals with more global features, so we expected that increasing the weighting on higher layer error would enhance global processing and prediction based on a wider spatial context. We compared the effect of the higher layer loss by setting λ_3_ = 0, 1, 100.

The second training condition is the state resetting in convolutional LSTM (ConvLSTM). PredNet produces predictions based on not only the current inputs but also the system's memory, which is stored as the internal state of ConvLSTM. Therefore, by interfering with this internal state, we can manipulate how long temporal context can be used for the prediction. Intuitively, the longer the available temporal context, the better the prediction. However, it has been reported that a longer temporal context can introduce instability to the system (Parisi et al., 2019). Also, the length of temporal contexts and the updating speed were reported to be related to ASD (Lieder et al., 2019).

We trained the network for each condition with different random seeds (*n* = 3), corresponding to the difference in the initial weights randomization.

To confirm that our networks were properly trained, we monitored the training loss throughout the learning process. The learning curves are provided in Supplementary Figure S6. While the loss values do not settle to very low levels due to the continuous (non-batch) video input, the curves confirm that learning has sufficiently converged.

### 2.4 Generating images for analysis

**Figure 2:**
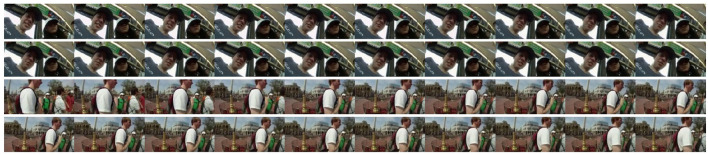
Examples of the inputs for the image generation for our analysis (images adapted from the First-Person Social Interactions Dataset (Fathi et al., 2012) and modified by Watanabe et al. (2018)). First, 20 consecutive frames of datasets were shown, and then 20 consecutive frames starting from randomly chosen time steps in the dataset were shown to the network.

To characterize the trained networks for each condition, we analyzed the behaviors during the prediction processes, which were described as follows:

Each prediction process consists of 60 time steps. In the first 20 time steps, we input 20 consecutive images from the dataset starting from a specific time point. Next, we input 20 consecutive images starting from the randomly chosen different time points in the dataset to observe the behavior upon the unpredictable abrupt scene change. In the last 20 timesteps, we conducted “extrapolation,” in which we used the generated image of the previous timestep $\hat{A_{0}}\left(t-1\right)$ as an input of the next timestep *A*_0_(*t*). The example of the first 40 input images is shown in Figure 2.

We repeated this prediction process 200 times for each trained network by selecting the first 20 images every 1,000 frames in the dataset.

To quantitatively support the visual inspection of generated images, we evaluated image quality and image statistics using the generated prediction sequences described above.

We computed PSNR and SSIM (as full-reference image-quality metrics), image sharpness using the variance of the Laplacian, brightness deviation, and saturation deviation. Brightness and saturation deviations were calculated in HSV color space as the absolute differences between predicted and target frames.

The primary analysis window was the post-change prediction period (*t* = 20–39), corresponding to the period after the abrupt scene change. Metrics were first averaged within each generated sequence and then summarized across random seeds. The results of this quantitative analysis are shown in Supplementary Figure S16.

### 2.5 Hilbert-Schmidt independence criterion (HSIC)

We analyzed the data acquired by the abovementioned prediction process using HSIC to quantify the top-down/bottom-up information flow during the prediction process.

HSIC quantifies the non-linear correlation between the two random variables (*X*, *Y*) and corresponds to approximations to the least-squares mutual information (Sugiyama and Yamada, 2012) in some cases. Here, we used the normalized HSIC (Wu et al., 2018), which is defined as


$$\begin{array}{l} \text{HSIC}\left(X,Y\right)=\frac{\text{tr}\left(KHLH\right)}{||HKH||||HLH||}, \end{array}$$

where $H=I-\frac{1}{n}11^{\text{T}}$, *K*: Gram matrix of *X*, and *L*: Gram matrix of *Y*. We chose a Gaussian kernel for the calculation of the Gram matrix.

We used this measure to quantify the top-down/bottom-up information flow in the following ways. As a measure of the top-down information flow for the prediction of layer *l*, we calculated the HSIC between the output of the layer Â_*l*_ and input from the higher layer, and the measure of the bottom-up information flow is quantified as the HSIC between the output of the layer Â_*l*_ and the input from the lower layer, which corresponds to the error of the lower layer.

The results below focused on the prediction at the bottom layer (layer 0). In this case the predicted image Â_0_ is a function of *E*_0_(*t*), *R*_1_(*t*), *R*_0_(*t*−1),


$$\begin{array}{l} {Â}_{0}\left(t\right)=f\left(E_{0}\left(t\right),R_{1}\left(t\right),R_{0}\left(t-1\right)\right). \end{array}$$

Therefore, we defined bottom-up HSIC and top-down HSIC as HSIC(Â_0_(*t*), *E*_0_(*t*)) and HSIC(Â_0_(*t*), *R*_1_(*t*)), respectively.

## 3 Results

We compare these trained networks across three aspects. The first part (3.1) characterizes the predicted images from each condition. The second part (3.2) presents the analysis of top-down/bottom-up information flow by HSIC. In the last part (3.3), we show the follow-up analysis on the trained network with reset conditions but different highest layer losses to investigate whether the increase in the weight of the highest layer loss enhanced the spatially global processing.

### 3.1 Characteristics of generated images from each condition

**Figure 3:**
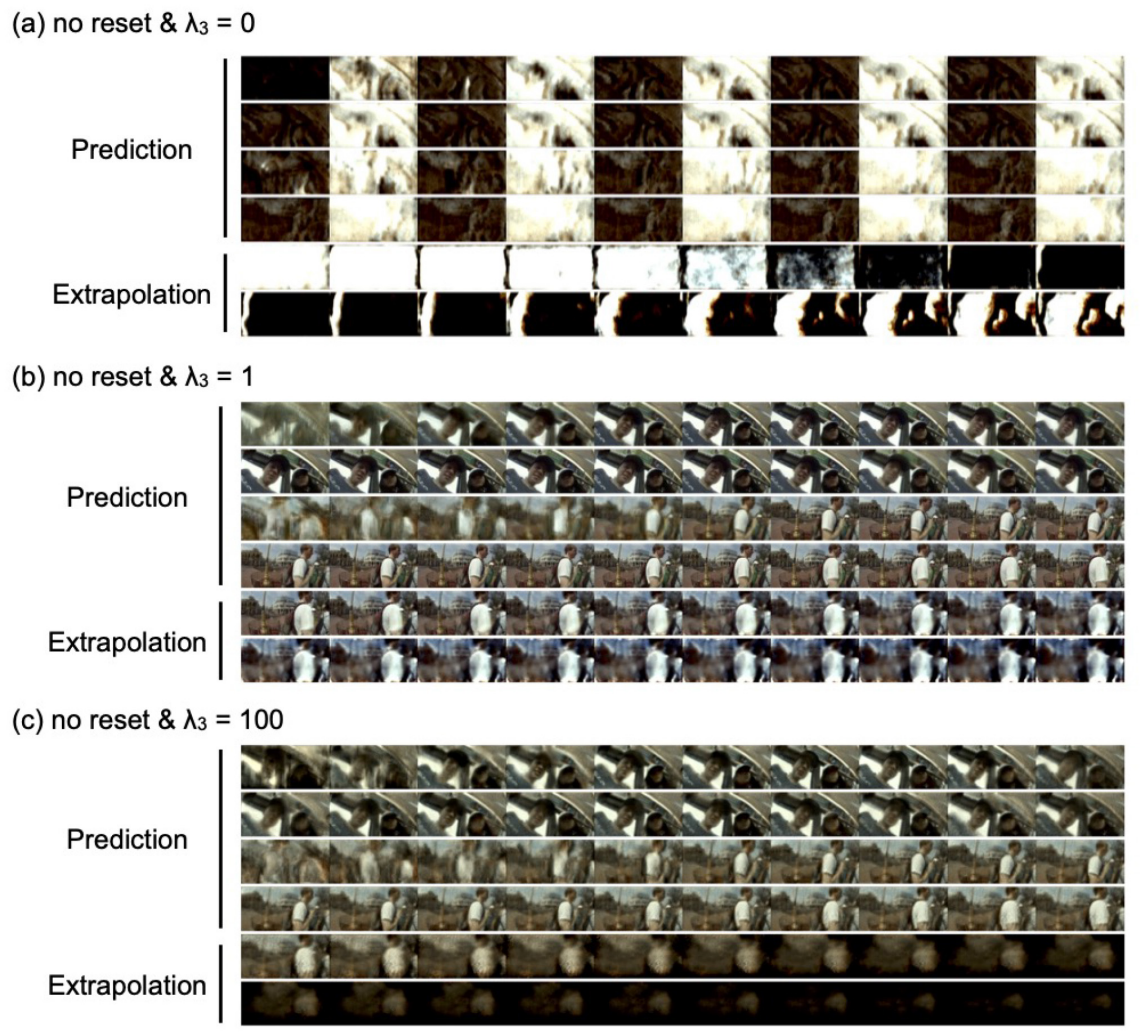
Examples of the generated images from “no reset & λ_3_ = 0” **(a)**, “no reset & λ_3_ = 1” **(b)**, and “no reset & λ_3_ = 100” **(c)** (images adapted from the First-Person Social Interactions Dataset (Fathi et al., 2012) and modified by Watanabe et al. (2018)). Each panel consists of 60 generated images. The first 40 images were generated by in putting the images in Figure 2, and the last 20 were generated by “extrapolation.”

**Figure 4:**
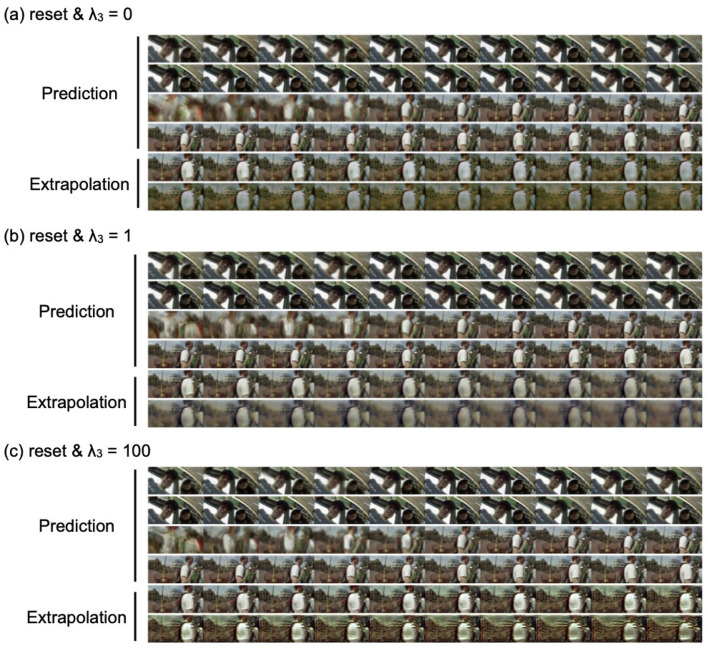
Examples of the generated images from “reset & λ_3_ = 0” **(a)**, “reset & λ_3_ = 1” **(b)**, and “reset & λ_3_ = 100” **(c)** (images adapted from the First-Person Social Interactions Dataset (Fathi et al., 2012) and modified by Watanabe et al. (2018)). Each panel consists of 60 generated images. The first 40 images were generated by inputting the images in Figure 2, and the last 20 were generated by “extrapolation.”

For our analysis, we generated predicted images from each trained network of each condition by the procedure described in 2.4. The examples of the generated images from each condition are shown in Figure 3 (no reset condition) and Figure 4 (reset condition). (More example images are shown in Supplementary Figures S9–S14).

To verify the robustness of these findings with respect to BPTT window length, we conducted additional experiments with BPTT = 40. As shown in Supplementary Figure S8, the reset condition continued to produce stable predictions without distortion, while the no-reset condition still exhibited distortions, though somewhat reduced compared to BPTT = 20, which were mitigated by higher layer loss.

**Figure 5:**
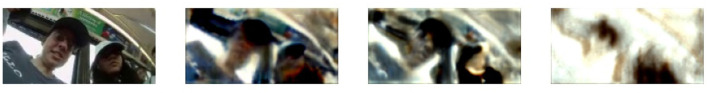
Examples of distorted images generated from the networks trained with no reset, λ_3_ = 0, and different random seeds (images adapted from the First-Person Social Interactions Dataset (Fathi et al., 2012) and modified by Watanabe et al. (2018)). From **Left to Right**: the input image, the predicted images (blurred, blurred and low chroma, blurred and high brightness).

The generated images from reset conditions were typically not distorted except for the initial period or immediately after the abrupt change at *t* = 20. On the other hand, we observed that prolonged visual distortion happened from the network trained with no reset conditions. (Figure 3, 4). In Figure 5, we show some typical examples (blurring, low chroma, and high brightness) of distorted images produced from no reset condition.

We also observed that these distortions were eased by adding higher layer loss (λ_3_ = 1, 100), which suggested that the higher layer loss stabilized the prediction even with no reset condition (Figure 3).

As an additional quantitative check, we computed PSNR, SSIM, image sharpness, brightness deviation, and saturation deviation during the post-change prediction window (*t* = 20 − 39; Supplementary Figure S16). The no-reset and λ_3_ = 0 condition showed the lowest PSNR and SSIM and the largest brightness and saturation deviations, whereas these indices were improved by positive higher-layer loss. These quantitative results support the visual observation that distortions were most pronounced in the no-reset condition without higher-layer loss and were mitigated by adding higher-layer loss.

### 3.2 Quantification of top-down/bottom-up information by HSIC

**Figure 6:**
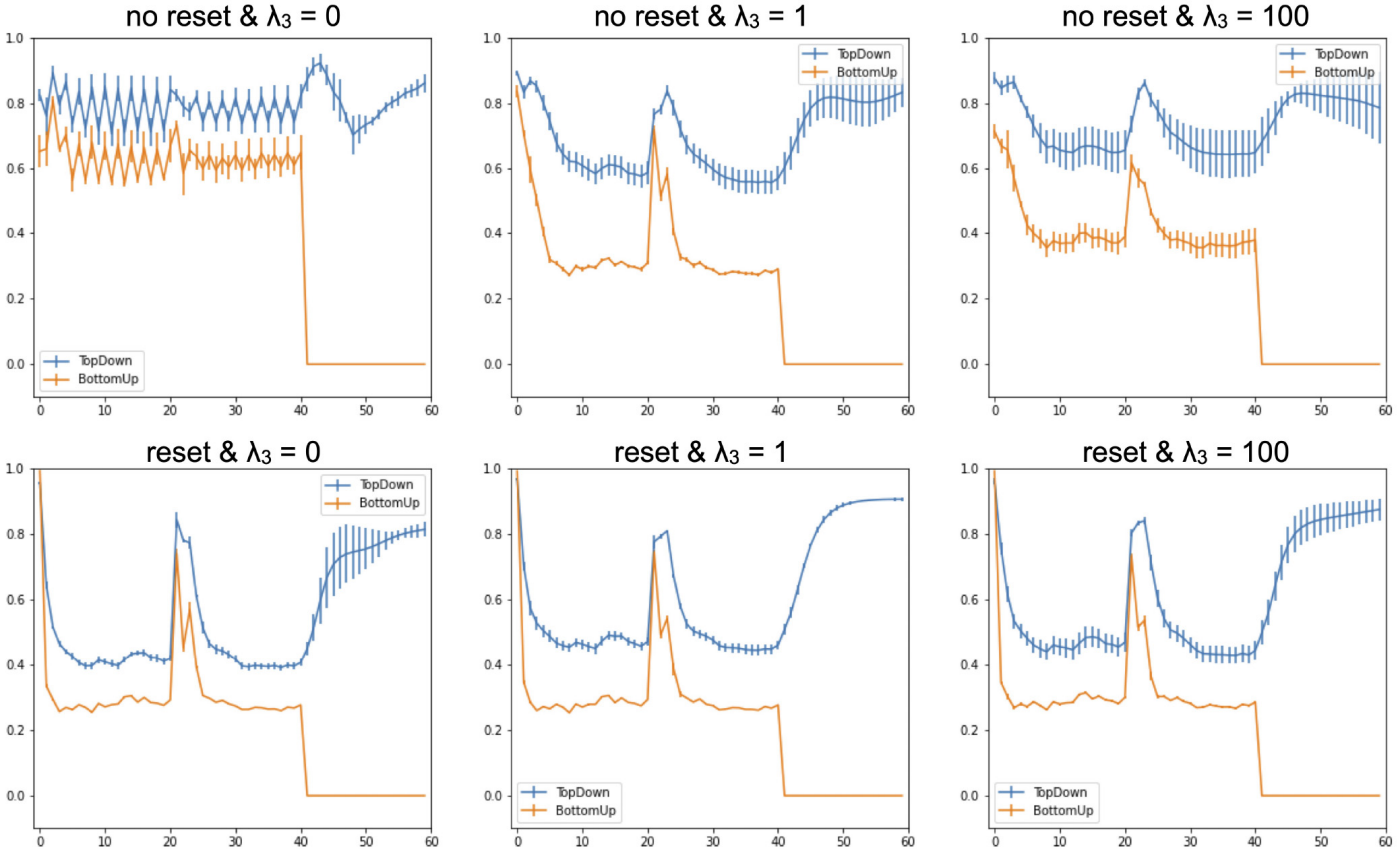
Characterization of the importance of top-down/bottom-up information to the output images using HSIC. The *x*-axis shows the time steps at the generation, from 0 to 39 corresponds to the image inputs like Figure 2, and 40–59 corresponds to extrapolation. From **top-left to top right**, “no reset and λ_3_ = 0,” “no reset and λ_3_ = 1,” “no reset and λ_3_ = 100,” “reset and λ_3_ = 0,” “reset and λ_3_ = 1” and “reset and λ_3_ = 100.”

Next, we quantified the top-down and bottom-up information used for the generated images by calculating HSIC(Â_0_(*t*), *R*_1_(*t*)) and HSIC(Â_0_(*t*), *E*_0_(*t*)), respectively. In Figure 6, we show the results from each training condition.

Typically, both top-down and bottom-up HSIC were high initially and gradually converged to a smaller value (0 ≤ *t* ≤ 20). Upon abrupt scene change (*t* = 20), both top-down and bottom-up HSIC rose again and then declined to specific values (20 ≤ *t* ≤ 40). Also, throughout 0 ≤ *t* ≤ 40, top-down information was usually higher than bottom-up information.

From *t* = 40, the images were generated by extrapolation, using the generated image as the input. In this case, there was no bottom-up information anymore (bottom-up HSIC = 0), and top-down HSIC increased (40 ≤ *t* ≤ 60).

In the case of no reset and λ_3_ = 0, unlike the typical behaviors mentioned above, both the top-down HSIC and bottom-up HSIC kept high during the prediction (0 ≤ *t* ≤ 40).

#### 3.2.1 Influence of higher layer loss in reset condition

**Figure 7:**
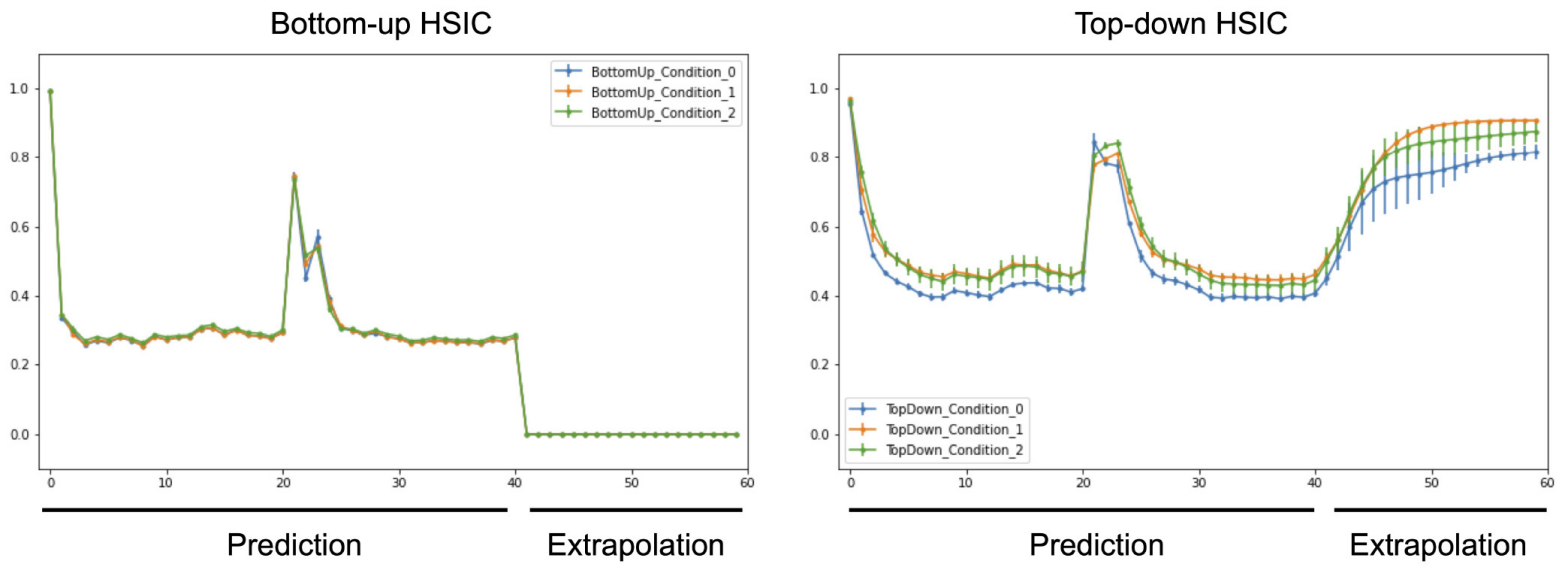
Comparison of bottom-up HSIC (**Left**) and top-down HSIC (**Right**) among the results from reset condition with different higher layer loss (λ_3_ = 0, 1, 100) (Overlaid).

To investigate the influence of the weight of higher layer loss λ_3_, we compared the results from the reset condition, which shared the same quantitative tendency described in the previous section (Figure 7).

Although the temporal dynamics were the same among different λ_3_, the top-down HSIC was higher when λ_3_ = 1, 100 than λ_3_ = 0, indicating that adding the higher layer loss enhanced the top-down information flow in the prediction process (Figure 7, Right). The top-down HSIC was also higher with positive λ_3_ during the extrapolation period. On the other hand, the bottom-up HSIC was not different among different λ_3_ (Figure 7, Left).

### 3.3 Higher layer loss strengthen the top-down information flow and global perception

In the previous section (3.2.1), we showed that adding higher layer loss λ_3_ during training increased the top-down information flow of the resulting network. To check whether this difference in the top-down information flow affected the generated images, we also conducted additional analysis on the trained network from the reset condition with different λ_3_.

#### 3.3.1 Global motion was well-captured in positive **λ**_3_ condition

**Figure 8:**
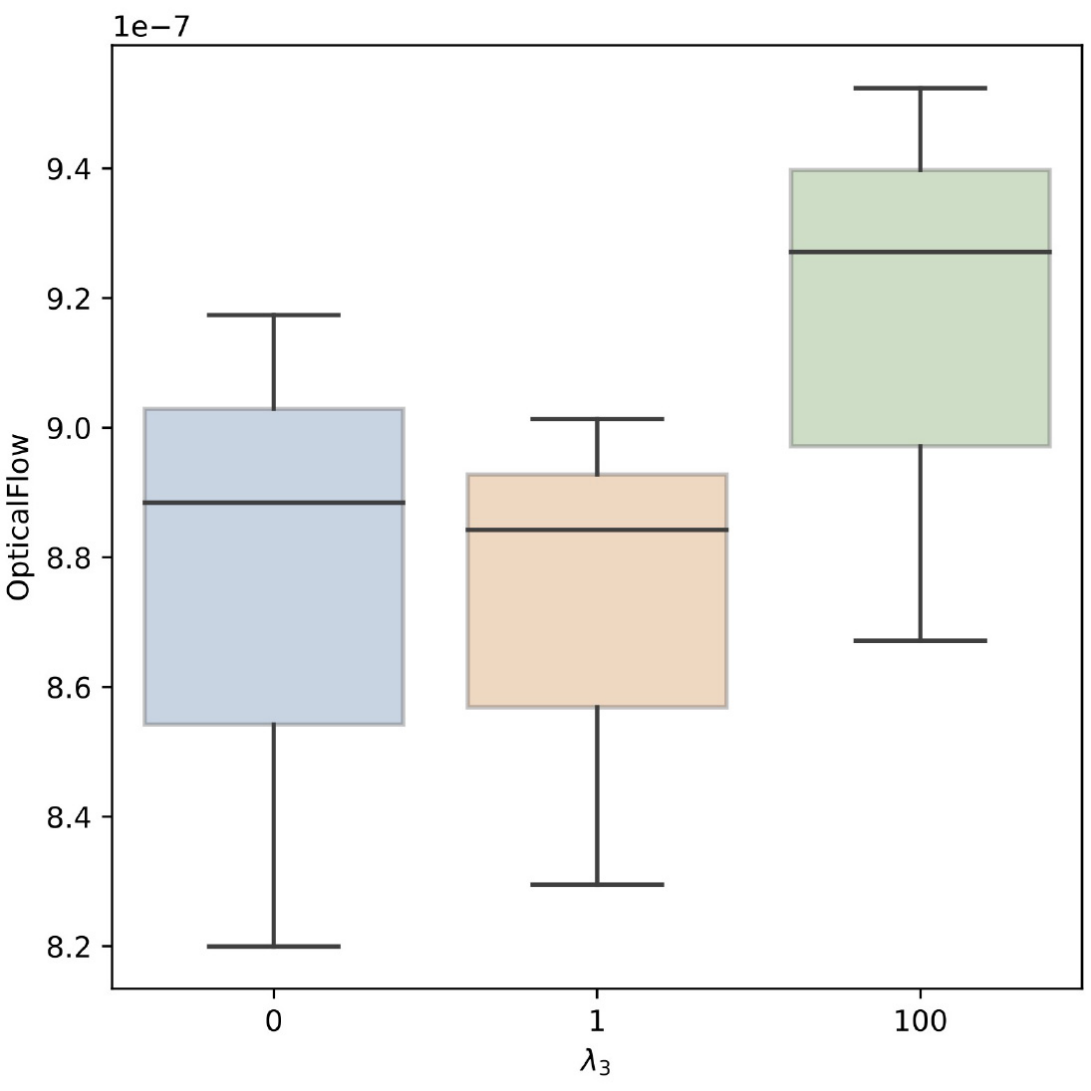
Comparison of global motion among different λ_3_ with reset condition. The degree of global motion was captured by the mean value of optical flow.

First, to check whether there were differences in replicating global motion in the predicted image sequences, we calculated the optical flow between the generated two successive frames to check whether each network can capture global motions. We found that the condition trained with high λ_3_ generated higher optical flow (Figure 8). By applying a generalized linear model, we confirmed that the optical flow was significantly enhanced in λ_3_ = 100 compared to λ_3_ = 0 (*p* < 0.05). This result shows that the generated image sequence from the positive λ_3_ condition captured the global motion well.

#### 3.3.2 Comparison of adaptation in global statistics

Because the higher layer encodes spatially coarse-grained information, we hypothesized that the increase in top-down HSIC leads to image predictions focusing more on global statistics, such as mean brightness and mean chroma.

**Figure 9:**
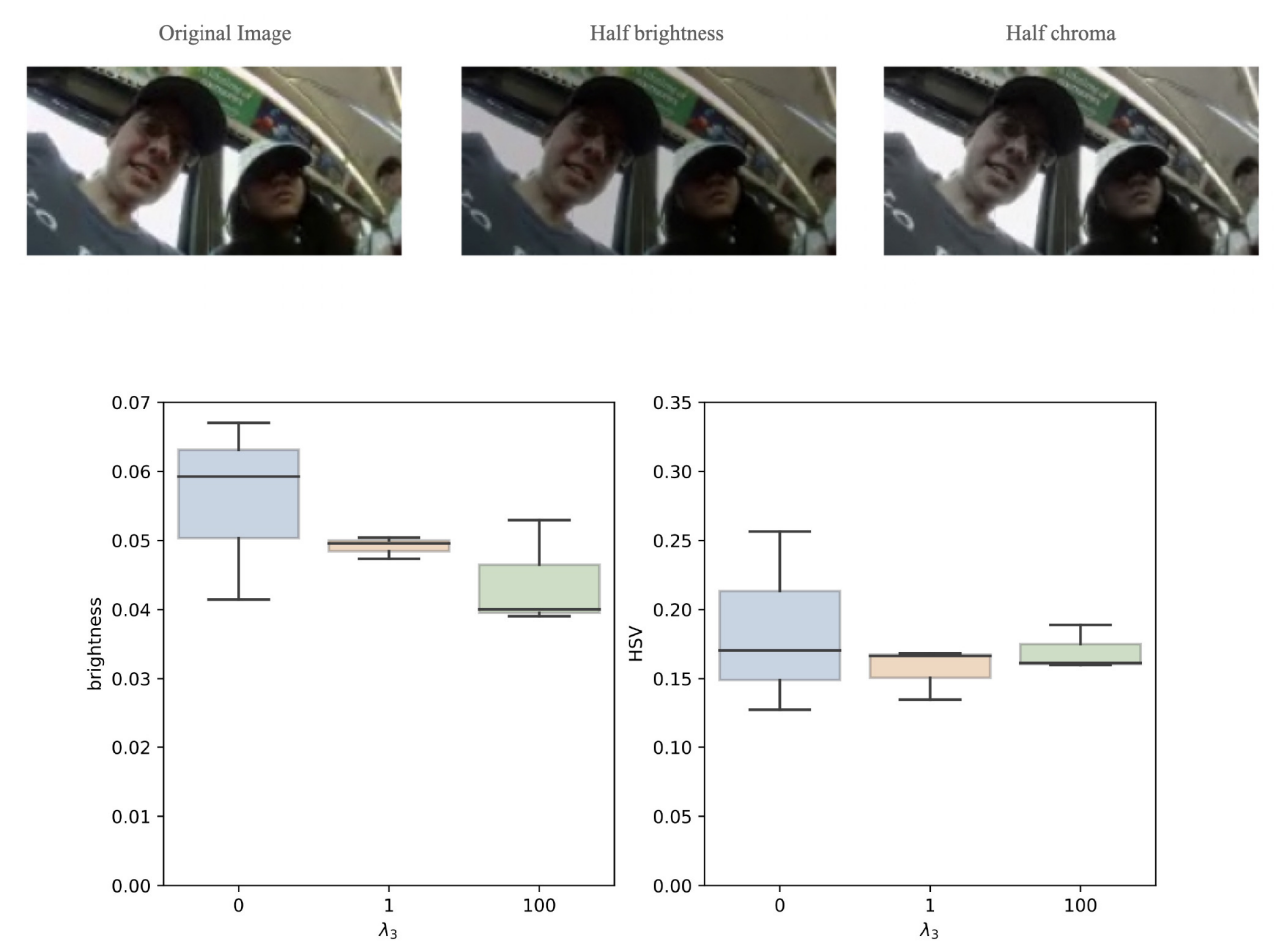
Comparison among networks trained with different λ_3_ and reset conditions. **Top:** the example of input images to investigate the behavior of each network, an original image, a half-brightness image, and a half-chroma image, respectively (images adapted from the First-Person Social Interactions Dataset (Fathi et al., 2012) and modified by Watanabe et al. (2018)). **Bottom:** the error of global statistics (brightness and chroma) of the predicted images right after the scene change among networks trained with different λ_3_ and reset conditions. Difference between the color hue (HSV) of the generated image and the input (half-color hue) images.

We prepared two different input image sets to test this and generate predictions. The first one (“Half Brightness Data”) was the image dataset used in 2.4 except with half brightness, which was prepared by multiplying 0.5 by each image pixel value, and the second one (“Half Chroma Data”), which was prepared by mixing the pixel value of the original images and their gray-scaled images (Figure 9). We generated image from these inputs by the trained network of reset condition and different λ_3_ and investigated how well these generated images captured the global statistics (mean brightness and mean chroma) of the input images. We found that the average brightness and chroma were captured well soon after the scene change with large λ_3_ (Figure 9). By applying GLM, we confirmed that the adaptation in average brightness was significantly enhanced in λ_3_ = 100 (*p* < 0.05) and average chroma in λ_3_ = 1, 100 (*p* < 0.001 and *p* < 0.05, respectively), compared to λ_3_ = 0.

## 4 Discussion

This paper uses PredNet to predict visual output, which can be distorted when trained under certain developmental conditions. Also, we show that the concept of top-down/bottom-up information can be applied to understand a high dimensional hierarchical predictive system beyond Gaussian approximation by using HSIC.

### 4.1 Visual distortions reproduced by PredNet

Our study demonstrated that specific developmental manipulations in PredNet can influence the stability and quality of visual predictions. The condition of state resetting, in which the internal state of ConvLSTM layers was periodically reset during training, was intended to simulate a reduced reliance on long temporal contexts. This condition contrasts with the no reset condition, where the internal state is continuously carried over, allowing the network to utilize extended temporal dependencies. The no reset condition produced significant visual distortions, such as blurring, low chroma, and high brightness, especially during transitions or extrapolations (Figure 3, 5). These findings align with the idea that prolonged temporal dependencies, while potentially beneficial, can introduce instability to predictive systems.

We introduced a higher-layer loss term λ_3_ in the objective function to counteract this instability during training. This manipulation was designed to enhance the influence of coarse-grained, top-down information processed in higher layers of the network. By increasing λ_3_, we observed a stabilizing effect on the network's predictions, with reduced visual distortions even under the challenging no reset condition (Figure 3). The results suggest that this added higher-layer loss promotes global feature integration, as evidenced by improved restoration of global motion and image statistics such as brightness and chroma (Figure 8, 9). In the context of machine learning, especially when training on short video clips, training with resetting conditions is natural because it simplifies the learning process by resetting the internal state between sequences. However, resetting internal states is unnatural in our daily perceptual experiences, which are continuous rather than segmented. Therefore, the no reset condition seems to model our continuous perception better as it allows experiences to flow seamlessly without being split into independent short temporal sequences. This kind of long-term learning is known to be vulnerable to catastrophic forgetting or interference (Parisi et al., 2019).

Here, we use the term temporal context in a broad sense, referring to temporal information carried through ConvLSTM internal states. More precisely, however, the reset/no-reset manipulation and the BPTT window length affect different aspects of temporal processing. The BPTT length determines how far gradients are propagated backward during learning, whereas the reset/no-reset manipulation determines whether recurrent internal states are periodically initialized or continuously carried over during the forward dynamics. Our additional BPTT = 40 experiment partially addressed this distinction: increasing the BPTT window reduced the distortions in the no-reset condition to some extent, but did not eliminate them, and the distortions were still mitigated by higher-layer loss. This suggests that the observed distortions were not simply due to the short BPTT = 20 window. Rather, continuous carry-over of internal states appears to be an important factor, potentially through accumulated recurrent-state drift or interference during generation. Because the learning curves indicated sufficient convergence, we do not interpret these distortions as a simple failure of training convergence, but as instability in the learned prediction dynamics under continuous state carry-over. A more systematic dissociation among reset interval, BPTT length, and hidden-state detachment remains an important direction for future work.

Our results suggest that enhancing top-down information flow enables stable perceptual predictions under such state-continuous training conditions while retaining state-mediated temporal information. Additionally, analysis using the HSIC revealed that networks trained with larger λ_3_ increased reliance on top-down information while maintaining consistent bottom-up contributions (Figure 6, 7). This balance allowed the network to process global features effectively and produce stable predictions, supporting the hypothesis that enhanced top-down flow can mitigate the effects of temporal instability in hierarchical predictive systems. It is known that ASD can accompany both visual distortions and deficits in global processing (Simmons et al., 2009; Robertson and Baron-Cohen, 2017), and our result can provide an integrative explanation for this based on the lack of enough top-down information flow.

### 4.2 Measurement of “precision” in general case

Our analysis primarily relies on a novel approach using HSIC, which enabled us to measure top-down/bottom-up information flow in the PredNet model. This measure is analogous to the precision (Friston, 2017; Lawson et al., 2014) in the hierarchical predictive processing system. The precision is formally defined as the inverse of the variance of the Gaussian distribution. In this case, the prediction can be calculated as the precision-weighted sum of the top-down/bottom-up information. However, this is only applicable under the Gaussian distribution assumption.

On the other hand, our analysis can apply to general cases without assuming low dimensional inputs or Laplace approximations. This method extends the applicability of the aberrant precision hypothesis to a broader class of computational models. (We also note here that precision is usually explicitly encoded in the previous models, but in our case, the value corresponding to precision was not explicitly encoded in the model, which suggests we might not need to design the precision control in the model, but this can emerge as a result of learning if the model and training data have some complexity.)

The relationship between precision and HSIC becomes explicit in an idealized scalar Gaussian-linear setting. Suppose that top-down and bottom-up signals are independent Gaussian variables, $X_{\text{TD}}\sim N\left(0,\pi_{\text{TD}}^{-1}\right)$ and $X_{\text{BU}}\sim N\left(0,\pi_{\text{BU}}^{-1}\right)$, and that the prediction is their precision-weighted average:


$$Y=\frac{\pi_{\text{TD}}X_{\text{TD}}+\pi_{\text{BU}}X_{\text{BU}}}{\pi_{\text{TD}}+\pi_{\text{BU}}}.$$

Then, the squared correlations between the prediction and each information source are


$$\rho^{2}\left(X_{\text{TD}},Y\right)=\frac{\pi_{\text{TD}}}{\pi_{\text{TD}}+\pi_{\text{BU}}},\;\rho^{2}\left(X_{\text{BU}},Y\right)=\frac{\pi_{\text{BU}}}{\pi_{\text{TD}}+\pi_{\text{BU}}}.$$

Because HSIC with a linear kernel reduces to the squared covariance, its normalized form reduces to the squared Pearson correlation coefficient in this scalar case (Kornblith et al., 2019),


$${\mathrm{nHSIC}}_{\text{lin}}\left(X,Y\right)=\rho^{2}\left(X,Y\right),$$

normalized linear-kernel HSIC corresponds to the normalized precision weights under this idealized Gaussian-linear setting. For example, when π_BU_ is fixed, nHSIC_lin_(*X*_TD_, *Y*) increases monotonically with π_TD_.

In the PredNet analysis, however, the Gaussian-kernel HSIC used in this study should not be interpreted as a direct estimator of precision. Rather, it measures non-linear dependence between prediction outputs and top-down or bottom-up variables. Although HSIC is useful as a dependence-based measure in high-dimensional non-linear systems, its sensitivity can depend on sample size, dimensionality, kernel choice, and the structure of dependence (Zhang et al., 2023). Therefore, we interpret HSIC as a precision-inspired dependence-based measure, rather than as a direct estimate of Bayesian precision.

From our HSIC analysis, we compared the importance of top-down/bottom-up information flow in the prediction process among different developmental conditions, as we discussed in the previous section. We also observed that the top-down HSIC is generally higher than the bottom-up HSIC. In the setup of PredNet, top-down information includes spatially coarse-grained data and internal states of the higher layers. Hence, this showed that the prediction is mainly based on the coarse-grained information.

Another information we can obtain from the analysis is the temporal change of these values. The typical temporal dynamics obtained in our experiment was, when the scene changed, both top-down/bottom-up information flow increased, which indicates that the prediction was mainly affected by the incoming information, and after this, both top-down and bottom-up information converged to smaller values, which suggests that the present sensory input does not fully determine the prediction, but more to do with internal states of the system.

Beyond analyzing individual models, our HSIC-based approach offers a principled framework for comparing different predictive coding architectures. Previous studies comparing models such as PredNet, Predictive Coding Networks (PCN) (Wen et al., 2018), and CortexNet (Ahmad et al., 2018) have primarily relied on task-specific performance metrics. However, direct comparison of their internal computational mechanisms has been challenging. The HSIC measure provides a model-agnostic way to quantify information flow patterns, potentially enabling systematic cross-architectural comparisons. This could help identify which aspects of predictive processing are architecture-specific vs. which represent more general computational principles.

### 4.3 Developmental condition and information flow

In this study, we compared the network with different training conditions, which we refer to as developmental conditions. This approach differs from previous research using computational models with explicitly encoded precision parameters. Precision can be directly manipulated in these models to observe its effects on behavior. However, PredNet does not explicitly encode precision; we can only indirectly manipulate the model's behavior by changing the training conditions. In this sense, our approach is not ideally suited for directly observing the influence of “aberrant precision.” Nonetheless, it is advantageous because it provides additional insight into the relationship between the developmental conditions and the resulting network structures. We note that previous research such as Idei et al. (2020, 2021); Takahashi et al. (2021); Idei et al. (2022); Soda et al. (2023) can be classified as this type of “developmental” approach.

Using this developmental approach, we confirmed that increasing the weights of higher layer loss λ_3_ during training (“development”) enhanced the top-down information flow. With this insight, we might predict what kind of developmental condition leads to the model with aberrant precision or even how we can interfere with the developmental process to ease the symptoms after the development.

### 4.4 Limitation of the current model

The main limitation of this paper came from the limitation of PredNet. PredNet is one of the few models based on a predictive processing framework that can conduct video prediction. However, PredNet's predictive ability is still limited. For example, Rane et al. (2020) pointed out that the primary strategy of PredNet is to copy the previous frame image or to blur the image, implying that PredNet is incapable of fully grasping the dynamics of the input images. Our results showed phenomenological variations but failed to cover many kinds of perceptual distortions, which might result from this limitation in predictive abilities.

Our experiment was initially motivated by the paper that reported that PredNet can reproduce illusory motion (Watanabe et al., 2018). However, these illusory motions might also be explained under the strategy described in Rane et al. (2020) because the illusory motion can be produced by just blurring the images (Supplementary Figure S5), suggesting that the illusory motion may be attributed to blurring occurring at the onset of extrapolation. As a side note, the training condition in Watanabe et al. (2018) corresponds to no reset condition with no higher layer loss in our case (Figure 3; Supplementary Figure S9), which produced highly unstable prediction and varied among different random seed sets at that training period (Supplementary Figures S1–S4).

Another limitation highlighted by Rane et al. (2020) concerns the observation that prediction errors in PredNet do not consistently decrease up the hierarchy. We also observed this pattern in our analysis (Supplementary Figure S7). This deviation from canonical predictive coding may reflect architectural constraints of PredNet. However, direct comparison of error magnitudes across layers may be complicated by the non-linear transformations and pooling operations that change the scale and nature of representations between layers.

While error-based analysis reveals these limitations, information-theoretic measures like HSIC provide a complementary perspective on hierarchical processing. HSIC assesses dependencies between layers independent of absolute scales, revealing patterns of information flow that may not be captured by prediction error alone. Our HSIC analysis shows that despite the architectural deviations from canonical predictive coding noted above, PredNet exhibits meaningful hierarchical information dependencies (Supplementary Figure S15).

We note that PredNet's architecture contains elements that deviate from canonical predictive coding formulations, as acknowledged by Lotter et al. (2020). Most notably, the model uses backpropagation through time (BPTT) for training, which is not biologically plausible. However, as in prior studies using PredNet and similar architectures, we view BPTT as a training tool rather than a model of biological learning mechanisms. Our focus is on the learned representations and network dynamics, which may still capture meaningful aspects of hierarchical predictive processing despite these implementation differences.

We also acknowledge the limitation of using a single dataset. The present experiments were conducted on a single long-duration first-person video dataset. Because our experimental design required continuous video streams to examine state continuity and hidden-state carry-over, full retraining and systematic validation across multiple standard video datasets, many of which consist of relatively short clips, were beyond the scope of the present study. Thus, the present results may partly reflect dataset-specific characteristics. Future work should test whether similar distortions emerge across diverse long-duration naturalistic video datasets and across different predictive architectures.

The other limitation resides in the measurement method, HSIC. This method is relatively efficient compared to simply calculating mutual information but still requires some computational burden when the dimension of each information flow is high. For practical usage, some approximation of this value might be necessary.

## 5 Conclusion

This study showed a novel approach to applying top-down/bottom-up information beyond the Gaussian distribution. By this method, we also directly measured the temporal dynamics of the relative importance of these information sources.

Our proposed methods open up the possibility of comparing different architectures using the same measures. The problem with the modeling study is that, though it can provide many varieties of models, usually, there are no proper ways to compare among different studies. Our method provides a way to bridge these different studies, which can lead to integrated views. This method also might clarify some missing links among different results obtained in neuroscience or psychiatry, such as our results suggesting the connection between the symptoms of visual distortions and global perception.

## Data Availability

The raw data supporting the conclusions of this article will be made available by the authors, without undue reservation.
