# Supplementary material for: Perceptual distortions in PredNet and quantification of top-down/bottom-up flow
Source: Front Comput Neurosci. 2026 Jul 7;20:1869519. doi: 10.3389/fncom.2026.1869519 (PMC13385115; doi:10.3389/fncom.2026.1869519)
Supplement: Supplementary file 1 [file Data_Sheet_1.pdf]

Supplementary Material

1 MOTION ILLUSION IN DIFFERENT CONDITIONS

In this section, we examined whether the motion illusion reported by Watanabe et al. (2018) was observed in our experimental conditions.

1.1 Methods

We evaluated the motion illusion of each trained network as follows. (This is based on the methods of Watanabe et al. (2018).)

We used the two static images (”rotating-snake” and ”non-rotating-snake ”) as input images. In the evaluation process, we first kept inputting one of these images for 20 steps, and we conducted extrapolation ten steps afterward.

We analyzed the degree of motion illusory by calculating optical flow using Farneback methods. From these values, we also calculated global rotation, calculated as the mean rotational angle of each point setting the center of the image as the center of rotation, and mean global rotation as the mean of the absolute value of each optical flow vector. We took a mean over $4 \leq t \leq 19$ for fixed input periods and $20 \leq t \leq 24$ for extrapolation.

1.2 Illusory rotation from published weights

First, we checked whether the illusory rotation was replicated in our optical flow analysis using the weight the authors published (Watanabe et al., 2018). We used two different weights. The first is the original weight, and the second is a newly published weight after modifying the image dataset(Watanabe, 2021).

The generated images and corresponding optical flows are shown in Fig.S1. We confirmed that the illusory rotation occurred only with a ”rotating-snake image” at the time of extrapolation ($20 \leq t \leq 24$), as reported in the original paper for both weights. Additionally, the original weight showed alternate rotation during an initial period ($4 \leq t \leq 19$) (Fig.S2).

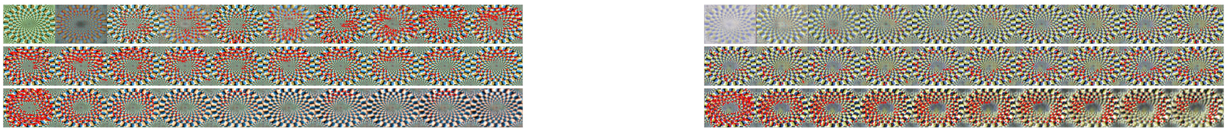

Figure S1. Examples of the generated images from published weights and corresponding optical flows. Right: Images generated by the initially published weight in Watanabe et al. (2018), Left: Images generated by the new version of the published weight.

1.3 Illusory rotation from newly trained weights

Next, we investigated the motion illusion of our newly trained weights. Here, we showed the results from two different training datasets; one is trained using the original dataset, and the second is trained by the modified dataset (cropped and skipped) used in the main text of our present paper. We trained the network with no resetting or resetting conditions with different random seeds ($n = 3$).

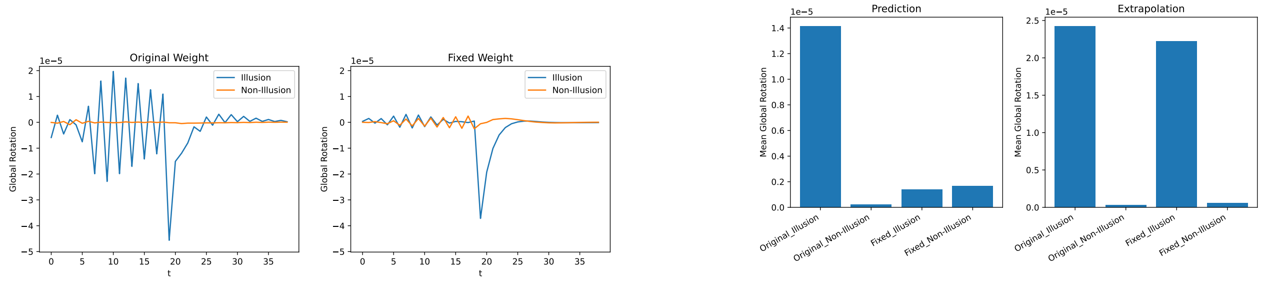

Figure S2. Optical flow results from each published weight (original and newly published). Left: Temporal changes in global rotations. This value was calculated as the mean angle of optical flows. (Blue: input with an illusory image, Yellow: input with a non-illusory image.) Right: Average of the mean strength of rotation for each condition. (Prediction: Average over prediction period ($4 \leq t \leq 19$), Extrapolation: Average over extrapolation period ($20 \leq t \leq 24$))

The examples of generated images are shown in Fig.S3.

We found that the results from no reset conditions showed varied optical flow values among different random seeds in the training, as expected from the instability reported in the main text. Still, the illusory rotation was observed at the beginning of extrapolation $(20 \leq t \leq 24)$ in most cases (Fig.S4, Top).

On the other hand, reset conditions showed less varied results and minor illusory rotation at $t = 20$, compared to the no resetting conditions (Fig.S4, Bottom).

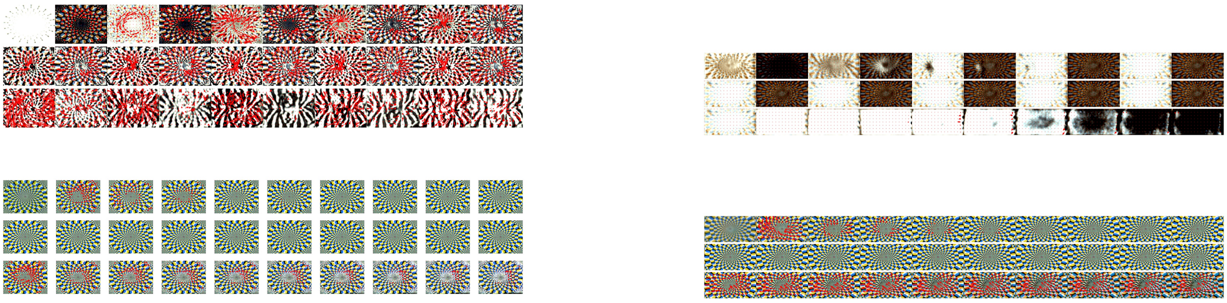

Figure S3. Examples of the generated images from newly trained weights and corresponding optical flows. Left: Images generated by the weight newly trained with the original dataset. (Top: no reset condition, Bottom: reset condition), Right: Images generated by the weight newly trained with the modified (cropped & skipped) dataset. (Top: no reset condition, Bottom: reset condition)

1.4 Gaussian blur can produce illusory rotation

From the inspection of these outputs, we noticed that the illusory rotation was often accompanied by blurring in the generated images. Here, we examined whether the blurring can produce apparent illusory motions.

For this purpose, we calculated the optical flow between the original images (”rotating-snake” and ”non-rotating-snake”) and blurred images generated by applying Gaussian blur. This blurring was applied not only to spatial dimensions but also to color channels.

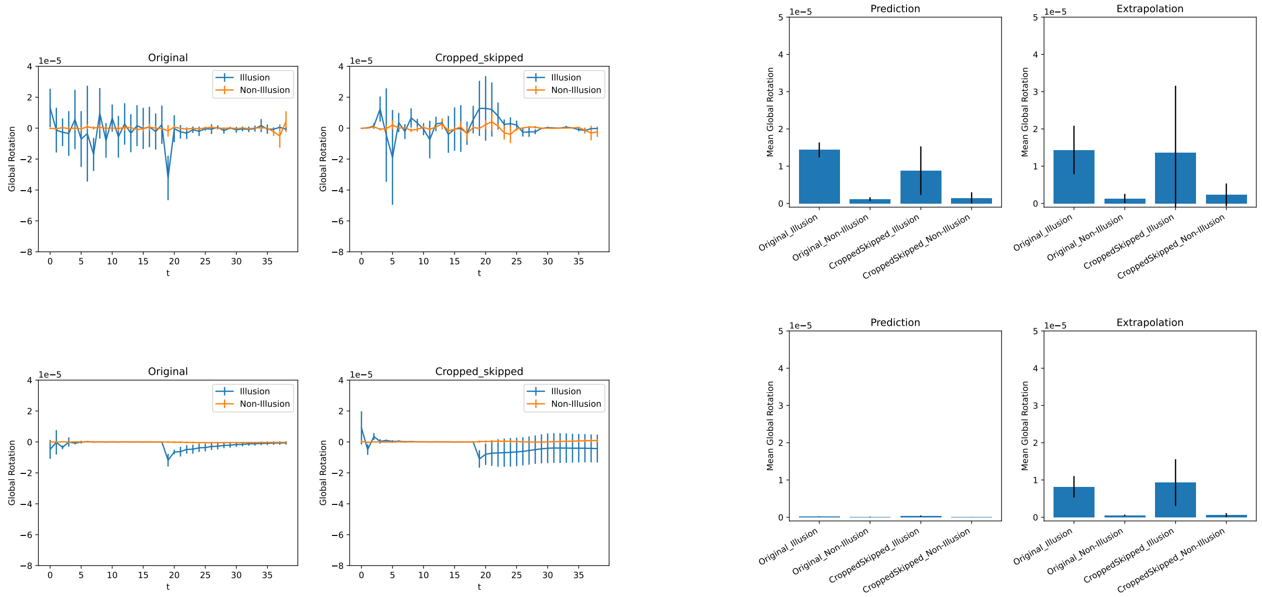

Figure S4. Optical flow results from each newly trained weight (Right column: trained with original dataset, Left column: trained with the modified dataset. Top: generated with no reset condition, Bottom: generated with reset condition). Left: Temporal changes in global rotations. This value was calculated as the mean angle of optical flows. (Blue: input with an illusory image, Yellow: input with a non-illusory image.) Right: Average of the mean strength of rotation for each condition. (Prediction: Average over prediction period ($4 \leq t \leq 19$), Extrapolation: Average over extrapolation period ($20 \leq t \leq 24$)).

As a result, we observed illusory rotation in the ”rotating-snake image” but not in the ”non-rotating-snake image” by blurring (Fig.S5), which indicates that the blurring can partly explain the illusory rotation observed in PredNet.

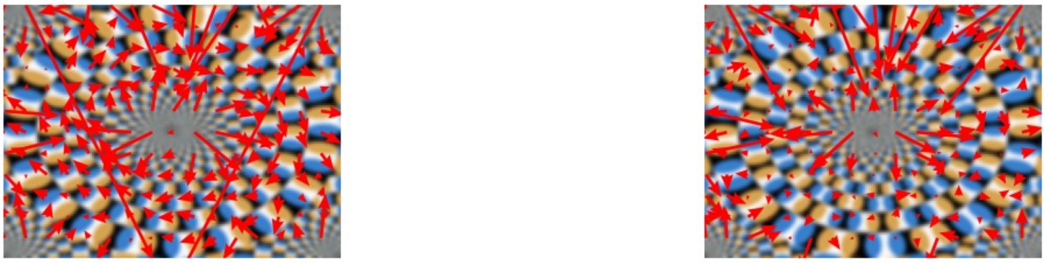

Figure S5. Results of optical flow analysis applied to blurred illusory image (Left) and non-illusory image (Right).

2 SUPPLEMENTARY ANALYSES AND TRAINING DETAILS

This appendix provides additional training details and analyses referenced in the main text, including learning curves (Fig. S6), prediction errors across layers (Fig. S7), effects of extended BPTT (Fig. S8), and additional examples of generated images.

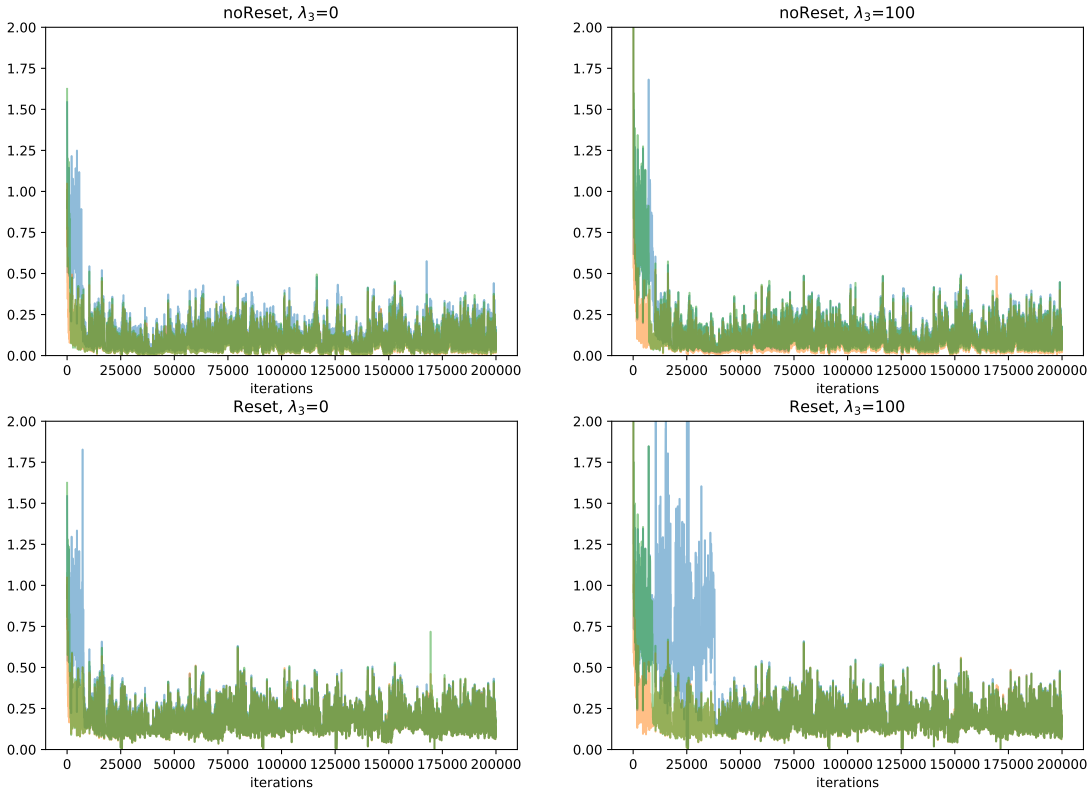

Figure S6. Training loss curves for all experimental conditions over 200,000 iterations. Each panel shows different training condition with $n = 3$ random seeds (overlaid).

2.1 Learning Curves

The training loss curves for all experimental conditions are shown in Fig. S6.

2.2 Prediction Errors Across Layers

Prediction errors at each layer are visualized in Fig. S7.

2.3 Effects of Extended BPTT

Results with BPTT extended to 40 steps are shown in Fig. S8. Distortions in no reset condition are reduced compared to BPTT=20 and mitigated by higher layer loss. Results confirm robustness across BPTT lengths.

2.4 More examples of generated images from different conditions

The examples of generated images from different weights (trained with different random seeds) are shown below (Fig.S9-S14).

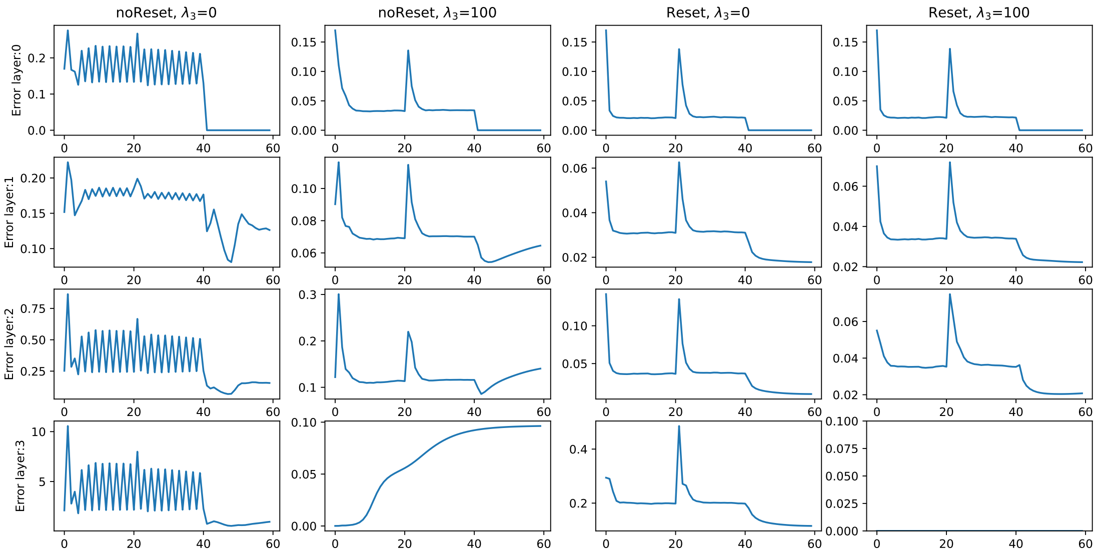

Figure S7. Mean prediction errors at each layer for each training conditions with the same random seed.

3 TOP-DOWN/BOTTOM-UP HSIC OF EACH LAYER

The calculation results of HSIC from each layer are shown in Fig.S15.

4 QUANTITATIVE EVALUATION OF VISUAL DISTORTIONS

To quantitatively support the visual inspection of generated images, we evaluated prediction quality and image statistics during the post-change prediction window ($t = 20$–39). The results are shown in Fig. S16.

REFERENCES

Watanabe, E. (2021). Sample Weight Models for Predictive Coding Deep Neural Network (Front. Psychol., 15 March 2018), and a Bug-fixed Version doi:10.6084/m9.figshare.11931222.v6

Watanabe, E., Kitaoka, A., Sakamoto, K., Yasugi, M., and Tanaka, K. (2018). Illusory motion reproduced by deep neural networks trained for prediction. *Frontiers in psychology* , 345

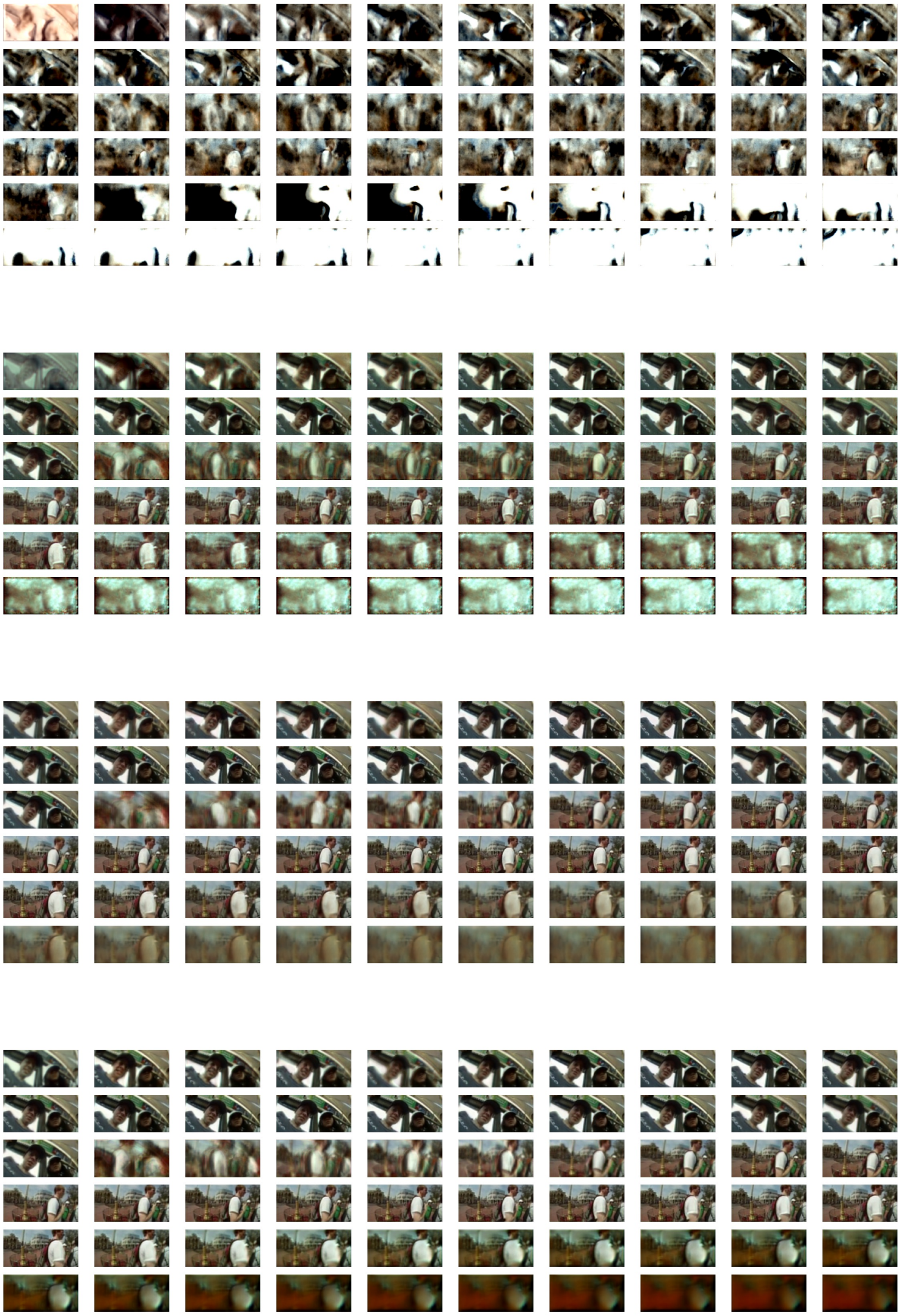

Figure S8. Effects of extended BPTT window (40 steps) on prediction quality. Rows show: (1) no reset & $\lambda_3 = 0$, (2) no reset & $\lambda_3 = 100$, (3) reset & $\lambda_3 = 0$, (4) reset & $\lambda_3 = 100$.

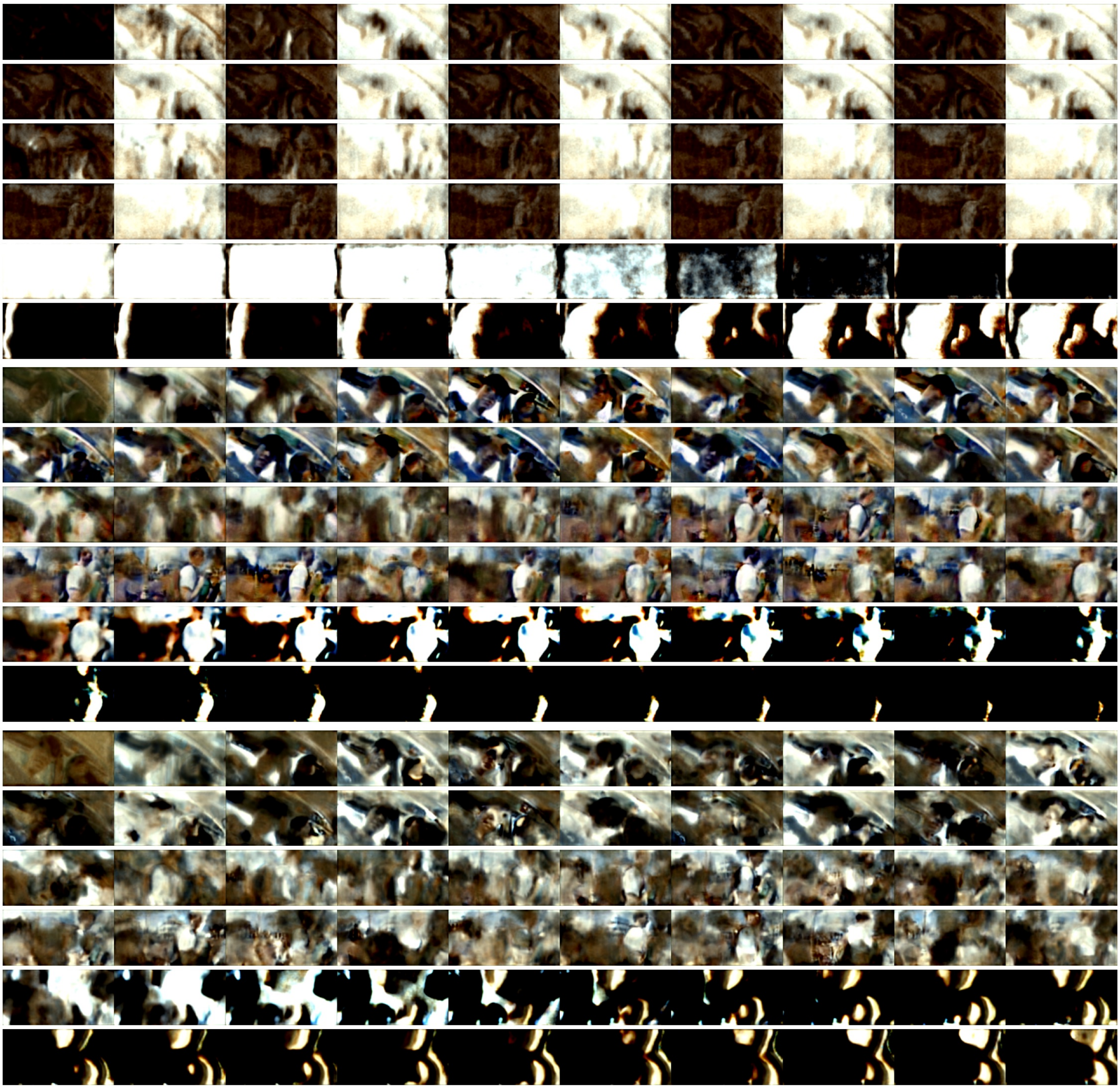

Figure S9. Examples of the outputs (no reset & $\lambda_3 = 0$ trained with three different random seeds)

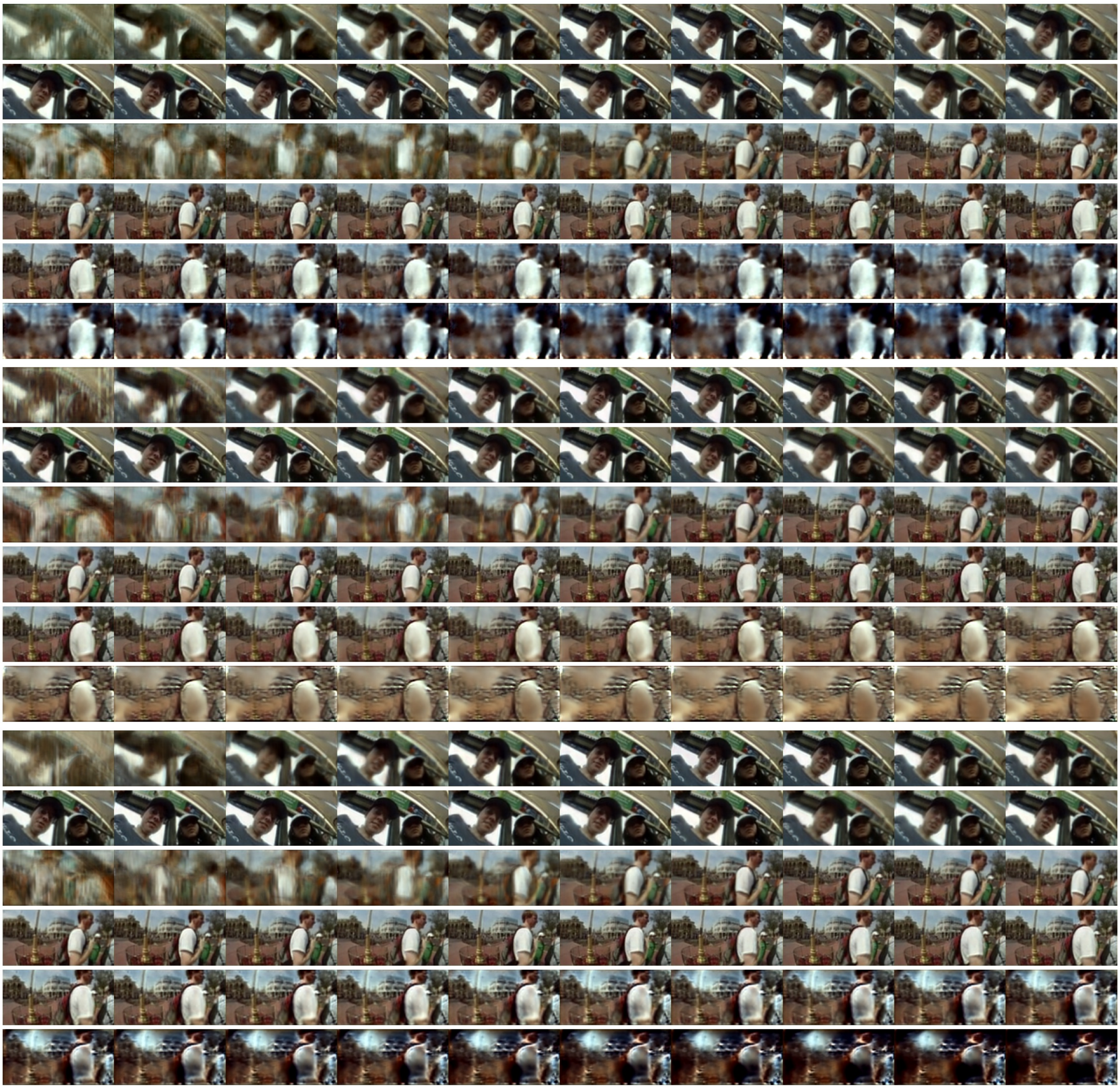

Figure S10. Examples of the outputs (no reset & $\lambda_3 = 1$ trained with three different random seeds)

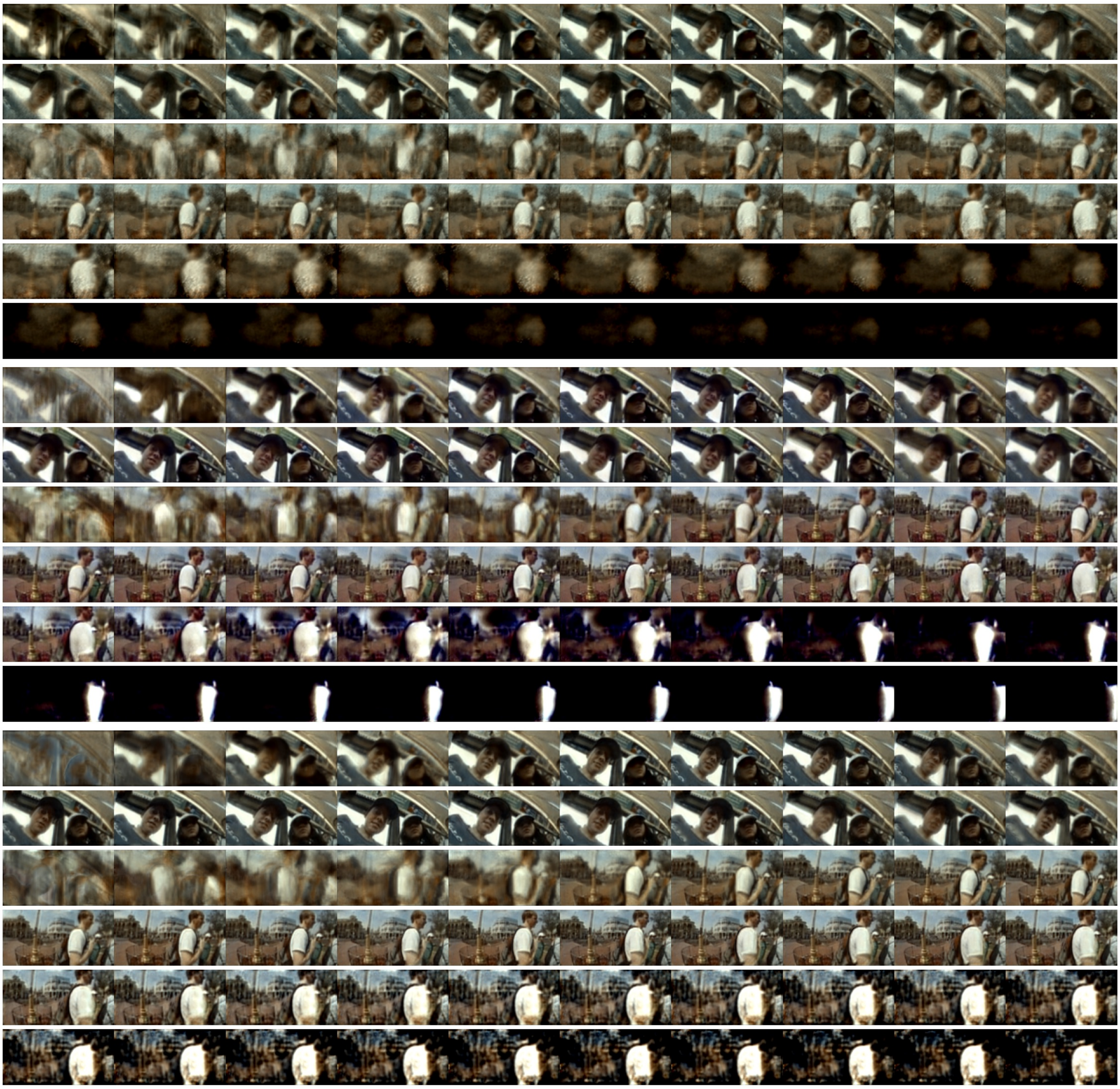

Figure S11. Examples of the outputs (no reset & $\lambda_3 = 100$ trained with three different random seeds)

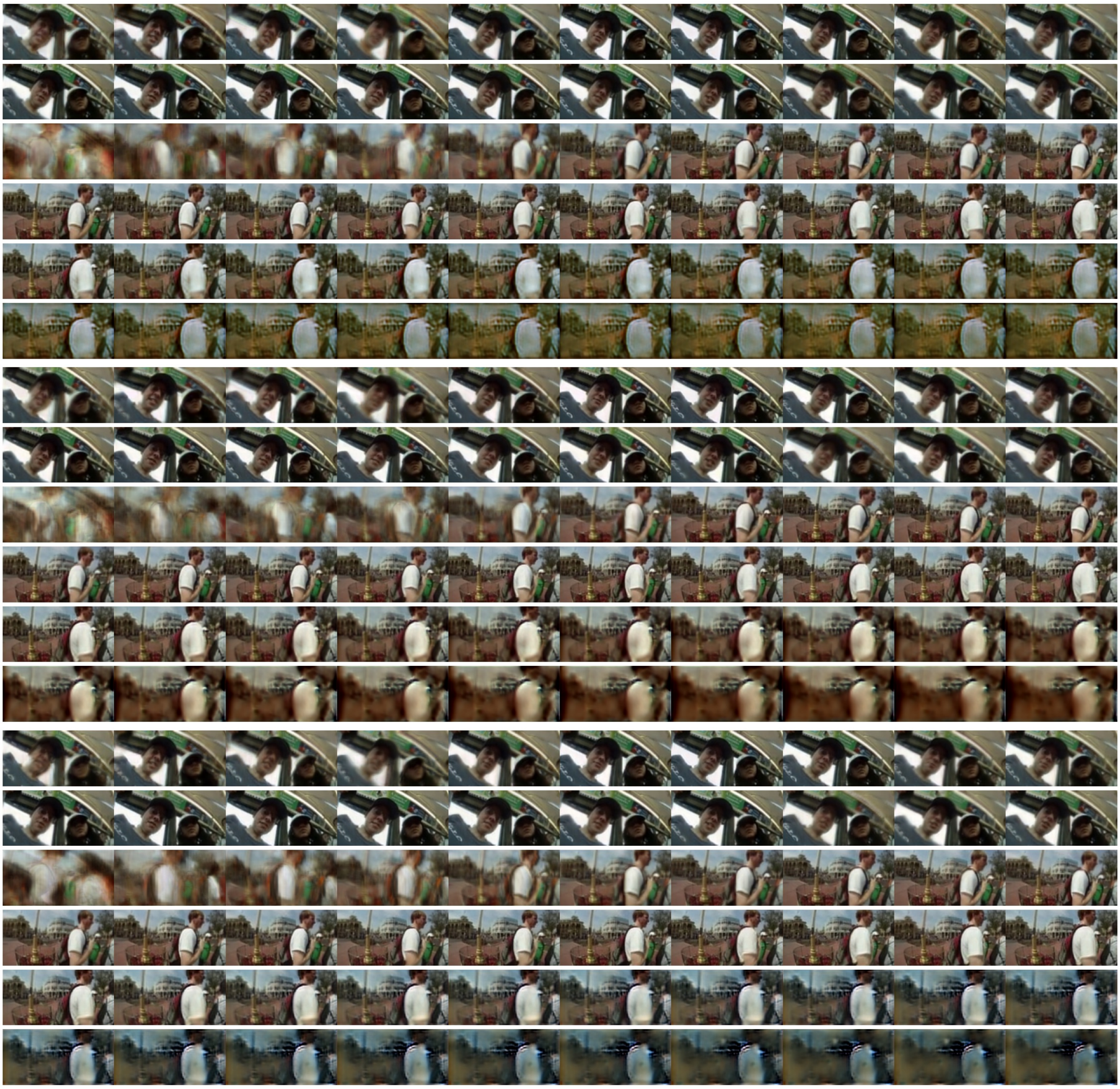

Figure S12. Examples of the outputs (reset & $\lambda_3 = 0$ trained with three different random seeds)

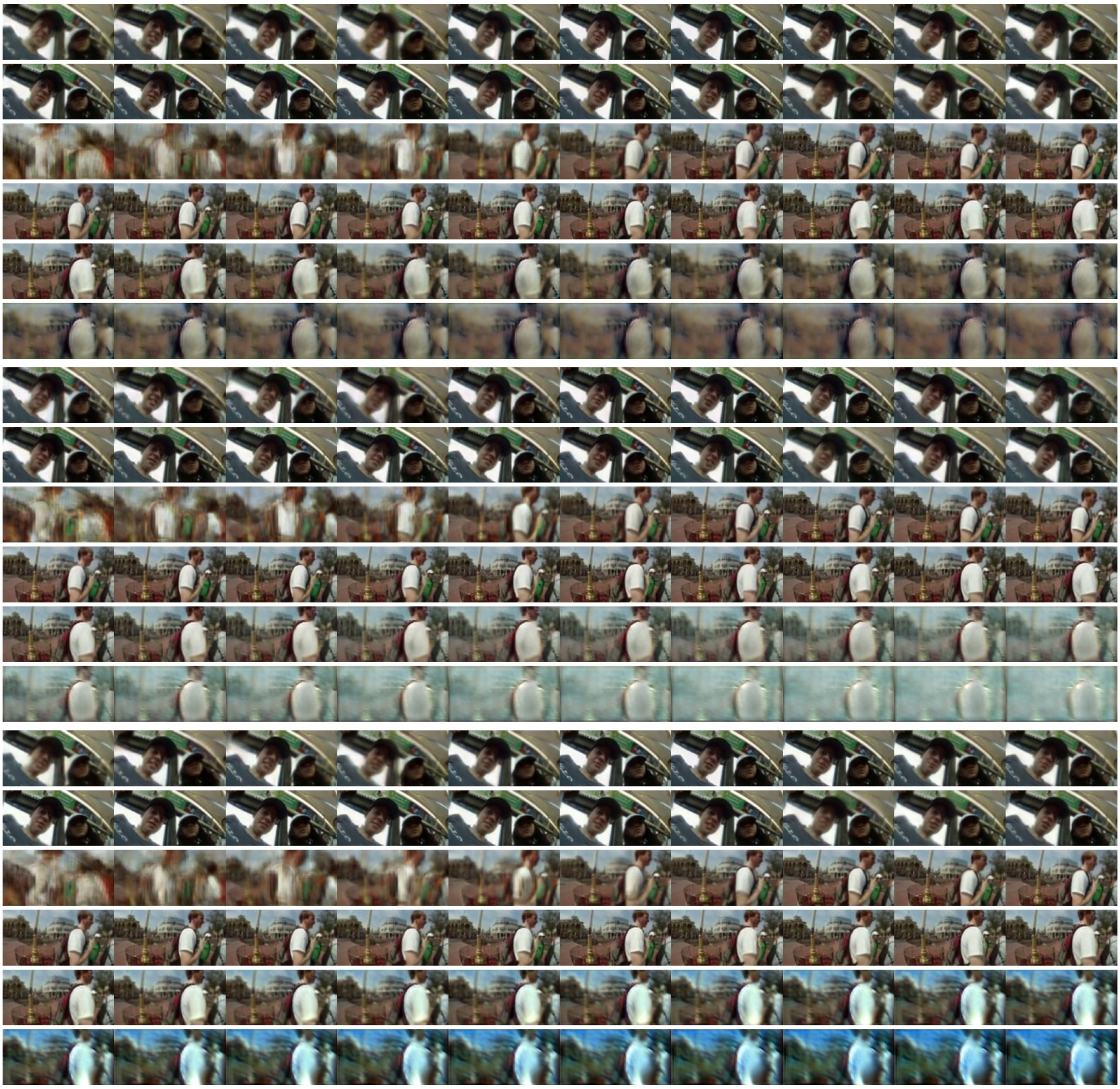

Figure S13. Examples of the outputs (reset & $\lambda_3 = 1$ trained with three different random seeds)

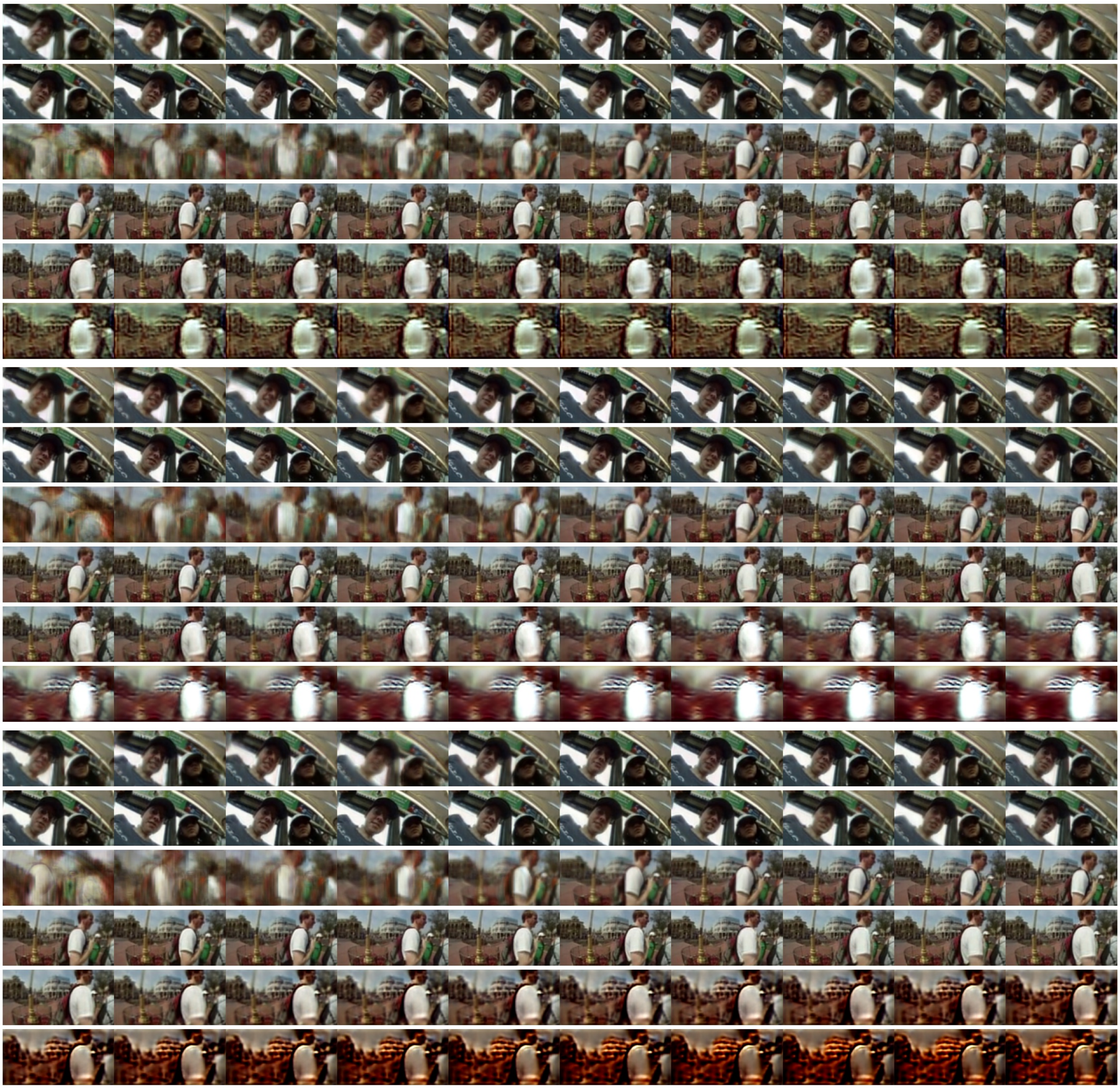

Figure S14. Examples of the outputs (reset & $\lambda_3 = 100$ trained with three different random seeds)

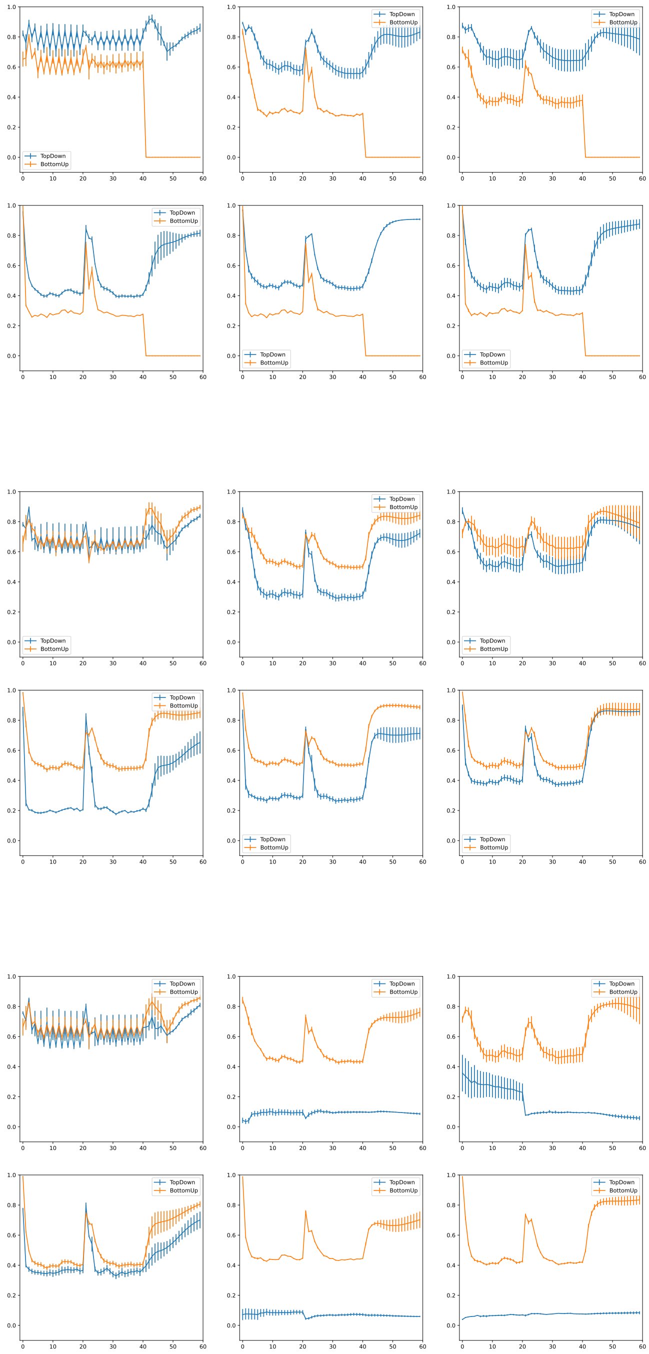

Figure S15. Characterization of top-down/bottom-up information for each layer (Top: $l = 0$,Middle: $l = 1$,Bottom: $l = 2$) by HSIC. From top-left to top-right in each panel, "no reset & $\lambda_3 = 0$", "no reset & $\lambda_3 = 1$", "no reset & $\lambda_3 = 100$", "reset & $\lambda_3 = 0$", "reset & $\lambda_3 = 1$" and "reset & $\lambda_3 = 100$".

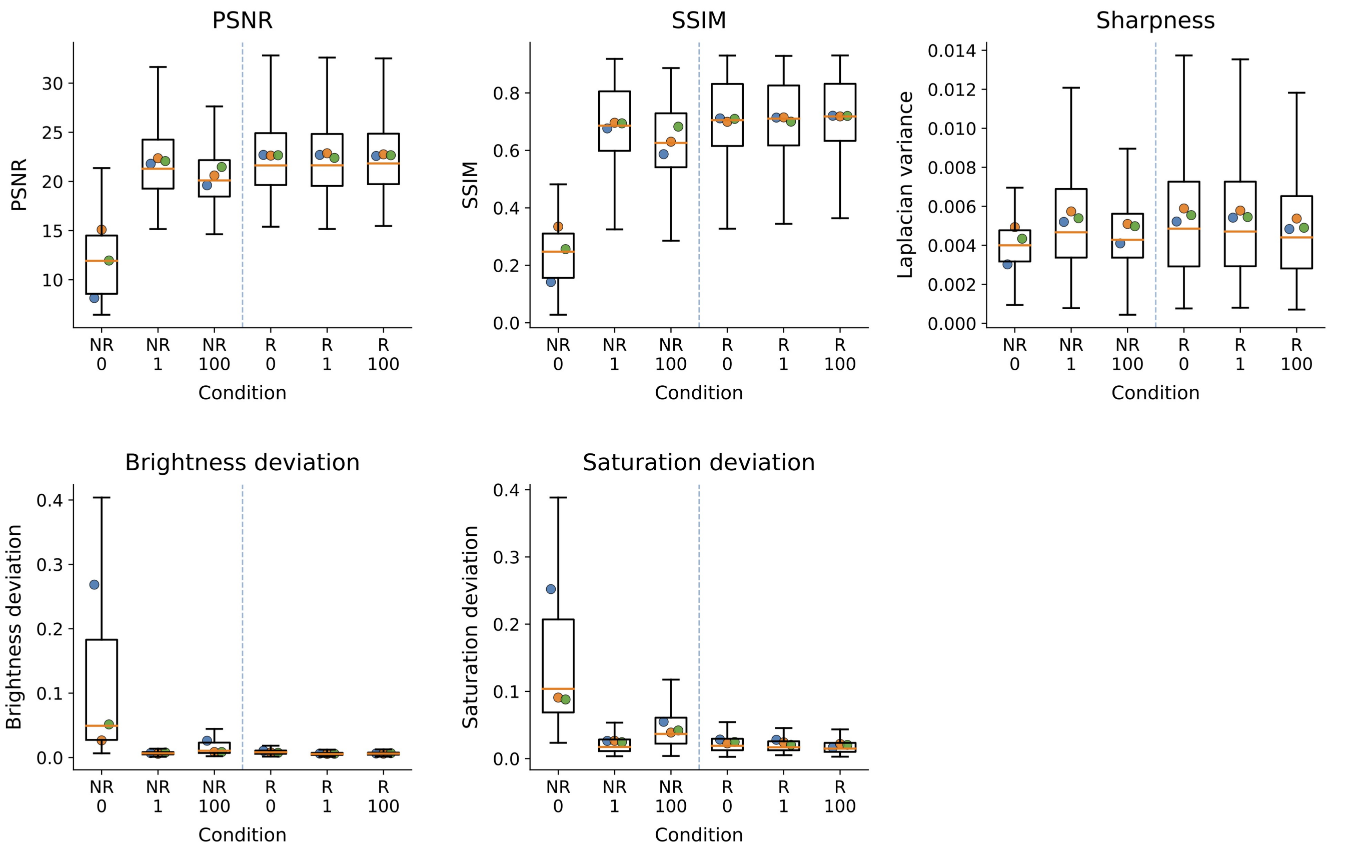

Figure S16. Quantitative evaluation of visual distortions during the post-change prediction window. (A) PSNR, (B) SSIM, (C) image sharpness measured by the variance of the Laplacian, (D) brightness deviation, and (E) saturation deviation were computed during $t = 20$–39. Boxes indicate distributions across generated sequences, and dots indicate seed-level means. NR, no reset; R, reset. Numbers indicate λ_3.
